# Characterization of canine tumor-infiltrating leukocyte transcriptomic signatures reveals conserved expression patterns with human osteosarcoma

**DOI:** 10.1007/s00262-025-03950-3

**Published:** 2025-02-11

**Authors:** Dylan T. Ammons, R. Adam Harris, Lyndah Chow, Steven Dow

**Affiliations:** 1 Flint Animal Cancer Center, Department of Clinical Sciences, College of Veterinary Medicine and Biomedical Sciences, Colorado State University, Fort Collins, CO 80523 USA; 2 Department of Microbiology, Immunology and Pathology, College of Veterinary Medicine and Biomedical Sciences, Colorado State University, Fort Collins, CO 80523 USA

**Keywords:** Canine (dog), Osteosarcoma, scRNA-seq, Transcriptomics, Cancer immunology

## Abstract

Immune cells play key roles in host responses to malignant tumors. The selective pressure that immune cells elicit on tumors promotes immune escape, while tumor-associated modulation of immune cells creates an environment favorable to tumor growth and progression. In this study we used publicly available single-cell RNA sequencing (scRNA-seq) data from the translationally relevant canine osteosarcoma (OS) model to compare tumor-infiltrating immune cells to circulating leukocytes. Through computational analysis we investigated the differences in cell type proportions and how the OS TME impacted infiltrating immune cell transcriptomic profiles relative to circulating leukocytes. Differential abundance analysis revealed increased proportions of follicular helper T cells, regulatory T cells, and mature regulatory dendritic cells (mregDCs) in the OS TME. Differential gene expression analysis identified exhaustion markers (LAG3, HAVCR2, PDCD1) to be upregulated in CD4 and CD8 T cells within the OS TME. Comparisons of B cell gene expression profiles revealed an enrichment of protein processing and endoplasmic reticulum pathways, suggesting infiltrating B cells were activated following tumor infiltration. Gene expression changes within myeloid cells identified increased expression of immune suppressive molecules (CD274, OSM, MSR1) in the OS TME, indicating the TME skews myeloid cells toward an immunosuppressive phenotype. Comparisons to human literature and analysis of human scRNA-seq data revealed conserved transcriptomic responses to tumor infiltration, while also identifying species differences. Overall, the analysis presented here provides new insights into how the OS TME impacts the transcriptional programs of major immune cell populations in dogs and acts as a resource for comparative immuno-oncology research.

**Supplementary Information:**

The online version contains supplementary material available at 10.1007/s00262-025-03950-3.

## Introduction

Systemic and local tumor immune responses have direct impacts on the clinical outcomes of cancer patients. For instance, overall survival and disease-free survival have been demonstrated to be positively correlated with effector T cell infiltration across multiple tumor types [1]. The infiltration of immune cells into a tumor is dependent on the early recruitment of cells to the tumor, and adequate antitumor adaptive immunity has proven to be fundamental for successful immunotherapy-based intervention [2, 3]. Although certain tumor microenvironments (TME) recruit and maintain enough immune cells to benefit from immunotherapy, other tumors, such as osteosarcoma (OS), are notoriously defined by poor immune cell infiltration [4]. The poor immune infiltration has contributed to the lack of response to immunotherapy in humans and dogs with naturally occurring OS [5]. Thus, there is a need to understand how the OS TME impacts tumor-infiltrating immune cells to facilitate the identification of immunotherapeutic targets.

Recent technological advances in single-cell RNA sequencing (scRNA-seq) have enabled the dissection of complex tissues and have aided in the discovery of previously unrecognized mechanisms of immune cell modulation in the TME [6]. The robustness of data integration approaches has also made it possible to compare gene expression profiles across tissue types [7–9]. Thus, normal tissues, such as circulating leukocytes, can be used as a point of reference to investigate TME-associated transcriptomic changes to infiltrating immune cells. Furthermore, canine OS is regarded as a valuable large animal model that enables the study of spontaneously occurring tumors and offers the opportunity to evaluate conserved TME components across species [10, 11]. As such this study aims to generate a canine-specific resource that documents TME-associated transcriptomic changes in immune cells, while also drawing parallels to human OS through side-by-side analysis of human OS scRNA-seq data.

In the present study we used two previously published canine scRNA-seq datasets consisting of tumor-infiltrating and circulating immune cells from dogs with OS to characterize the transcriptomic responses to infiltration [12, 13]. Following identification of transcriptomic responses within major canine immune cell populations, previously published human scRNA-seq data was analyzed to identify conserved and divergent aspects of transcriptomic responses. The purpose of the study was to investigate how the canine OS TME impacts immune cell gene expression profiles, generate gene signatures that capture transcriptomic changes within each immune cell type, and characterize genes as conserved or divergent relative human OS.

## Materials and methods

### Data acquisition, read mapping, and quantification

Raw FASTQ data generated from Ficoll-Paque prepared canine whole blood and treatment naïve OS tumor biopsies using the 10x Genomics Chromium platform were obtained from NCBI GEO (GSE225599 and GSE252470) [12, 13]. The raw data were aligned to the canine genome (CanFam3.1 Ensembl, release 104) and count matrices generated using a Cell Ranger analysis pipeline (version 6.1.2, 10x Genomics) as previously described [12]. Annotated datasets from the primary reports were obtained from GSE225599 and from Zenodo (https://doi.org/10.5281/zenodo.10666968).

### Data filtering and integration

For each sample, the count matrix was imported into R using the *Read10X* function then converted to a Seurat object using the *CreateSeuratObject* function [14]. Dead and poor-quality cells were filtered out by only retaining cells that met quality control thresholds. For the tumor dataset thresholds used were: 200 < nFeature_RNA < 5500, percent.mt < 12.5, and 100 < nCount_RNA < 15,000. For the blood dataset thresholds used were: 200 < nFeature_RNA < 3500, percent.mt < 20, and 500 < nCount_RNA < 20,000. After initial filtering, DoubletFinder was used to remove putative cell doublets [15]. Samples within each tissue were integrated into separate objects using a SCTransform normalization protocol and canonical correlation analysis (CCA) integration workflow. During integration the percent mitochondrial reads was used as a latent variable in a linear regression framework to minimize the impact of mitochondrial reads on dimension reduction and integration. After each tissue type had all samples integrated into its respective object, previous cell type annotations were transferred and any cells lacking annotation were removed. To focus analysis on cell types that are found in circulation and in tumors, we excluded non-immune cells from the tumor dataset. Furthermore, mast cells, osteoclasts, IFN T cells, and macrophages were also excluded from analysis due to a lack of a circulating counterpart. The blood dataset was filtered to exclude eosinophils, double negative T cells, γδ T cells, IFN signature CD4 T cells, basophils, and CD34^+^ unclassified cells. All filtered samples were then integrated into one object using the same approach applied to integrate individual tissues. The top 2500 variably expressed features were used as integration anchors then unsupervised clustering was completed. Ideal clustering parameters were identified using the R package clustree [16]. Dimension reduction and visualization was then completed, and the data were presented using two-dimensional, nonlinear uniform manifold approximation and projection (UMAP) plots.

### Subcluster analysis

For each of the major immune cell type populations, the dataset was subset to include only cells from one major population. The subset dataset was then used to identify new highly variable features, then the data were re-integrated and dimension reduction was repeated as described for the full dataset.

### Cell classification

Cell annotations, as previously reported, were transferred to the integrated dataset using the unique cell barcodes associated with each cell [12, 13]. Unsupervised clustering was completed, then the composition of cell types (based on the transferred classifications) within each cluster was examined. For clusters in which one cell type predominated, the label was directly transferred. When conflicting cell types fell within a cluster, a new cell identity was assigned to capture the cells present in the unsupervised clusters identified in the current study. The gene signatures of each cluster identified in this manuscript, as determined using the *FindAllMarkers* function in the Seurat package (test.use = “wilcox”, only.pos = TRUE), are provided as supplemental data.

### Feature visualization

Feature expression was visualized using feature plots. Selected features were chosen based on the identification of a feature to be differentially expressed when contrasting tumor-infiltrating and blood leukocytes. Feature plots depict normalized expression for each feature and are presented on variable scales. When visualizing expression between tumor and blood leucocytes in a UMAP embedding, tissues were down sampled to obtain equal representation of each tissue.

### Cell abundance analysis

All cell abundance comparisons were made using the percentage of total cells in the subset being analyzed. To make statistical inferences on changes in cell abundance two-sided Wilcoxon Rank Sum tests were used. Differences in cell abundances were discussed as over-/under-represented or unique. Relative abundance differences were classified as over-/under-represented if *P* value < 0.05 and | log2(Fold change) |< 3 when comparing between the two tissues sources. The term “unique” was reserved for changes in which *P* value < 0.05 and | log2(Fold change) |> 3. The classification scheme was designed to identify unique cell types based on the idea that cell types with low to no representation will result in an exaggerated log2(Fold change), and in turn pass the high-end cutoff.

### Differential gene expression analysis

Differential gene expression (DE) analysis was completed using pseudobulk conversion followed by a DESeq2 pipeline [17]. Prior to running DESeq2, low abundance features, defined as features that had less than 10 cells with raw counts greater than 1 across all cells sampled, were filtered out. For analysis comparing gene expression between tumor-infiltrating and blood leukocytes, *P* values were determined by testing the null hypothesis that | log2(Fold change) |< 0.58. Features were then considered to be significantly differentially expressed if the adjusted (FDR) *P* value was less than 0.01. Any subsequent gene set enrichment analysis (GSEA) was completed using lists of upregulated genes and the clusterProfiler package [18]. Gene ontology, Reactome, and ImmuneSigDB gene sets were used [19], and terms were considered enriched if they achieved an adjusted (FDR) *P* value less than 0.05. For ImmuneSigDB analysis the pathways included were filtered to include only terms relevant to the cell type being studied. The *AddModuleScore* function from the Seurat R package was also used to investigate enrichment of predefined cell type gene signatures (Supplemental Table 1).

### Identification of tissue signatures and removal from analysis

After completing DE analysis for each major cell population, we observed a bias for tumor-infiltrating immune cells to have increased expression of extracellular matrix associated features. Given previous reports documenting that the release of mRNA during sample processing and subsequent incorporation into cell droplets can result in confounding background tissue signatures, we devised a strategy to identify and remove features associated with background tissue signatures [9, 20]. To accomplish this, we completed differential expression analysis within each major immune cell population, then evaluated the features for consistent differential expression across all cell types. This revealed 46 features to be upregulated and four to be downregulated (TXNIP, PPBP, STK38, MITD1) across all major tumor-infiltrating cell types (Supplemental Table 2). GSEA of the 46 features revealed associations with extracellular matrix pathways, further suggesting that the gene signatures originated from non-immune cells and represented background noise (Supplemental Fig. 1) [20]. For tissue signature identification, we considered features with a *P* value less than 0.05 to be significant when testing the null hypothesis that | log2(Fold change) |< 0.58. We excluded the 46 tumor tissue-associated features, the 4 blood associated features, and a list of 108 platelet associated features (Supplemental Table 3), then repeated DE analysis as described above [12].

### Human–Canine transcriptomic response homology analysis

Raw scRNA-seq data from 6 treatment-naive human OS samples and 4 healthy human Ficoll Paque prepared whole blood samples were obtained from NCBI GEO database accession GSE162454 and GSE149689, respectively (Supplemental Table 4) [21, 22]. The samples were aligned to the human genome (GRCh38; GENCODE v44/Ensembl 110 annotations) and count matrices were generated using Cell Ranger as completed for the canine data. The count matrices were loaded in as Seurat objects and previously published cell type annotations were loaded to group cell types by the same major classifications identified in the canine dataset. Neutrophils and B cells did not have adequate representation, so only CD8 T cells, CD4 T cells, dendritic cells, and monocytes were included in homology analysis. Canine gene symbols were converted to human gene symbols retaining only genes with a 1:1 orthologue. Genes which had detectable counts in both of the full human and canine datasets were used to complete DE analysis using the same approach described above. After completing DE analysis in each species, genes were classified as conserved, ambiguous, divergent, or not significantly differentially expressed. Conserved genes were those differentially expressed in at least one species and both species had the same direction of log2(fold change). Ambiguous genes were those differentially expressed in one species, but not expressed (or too few counts to pass the low abundance filter) in the other. Divergent genes were classified as those that were differentially expressed in at least one species and the log2(fold change) was in different directions.

#### Data and software availability

A project specific GitHub page containing all analysis code and software versions used to analyze the data presented in this manuscript is available at https://github.com/dyammons/canine-blood-VS-tils-scrna. The annotated dataset is available for browsing through the UCSC Cell Browser at https://canine-tils-blood.cells.ucsc.edu [23].

## Results

### Summary of the integrated canine blood and tumor-infiltrating immune cell dataset

**Fig. 1:**
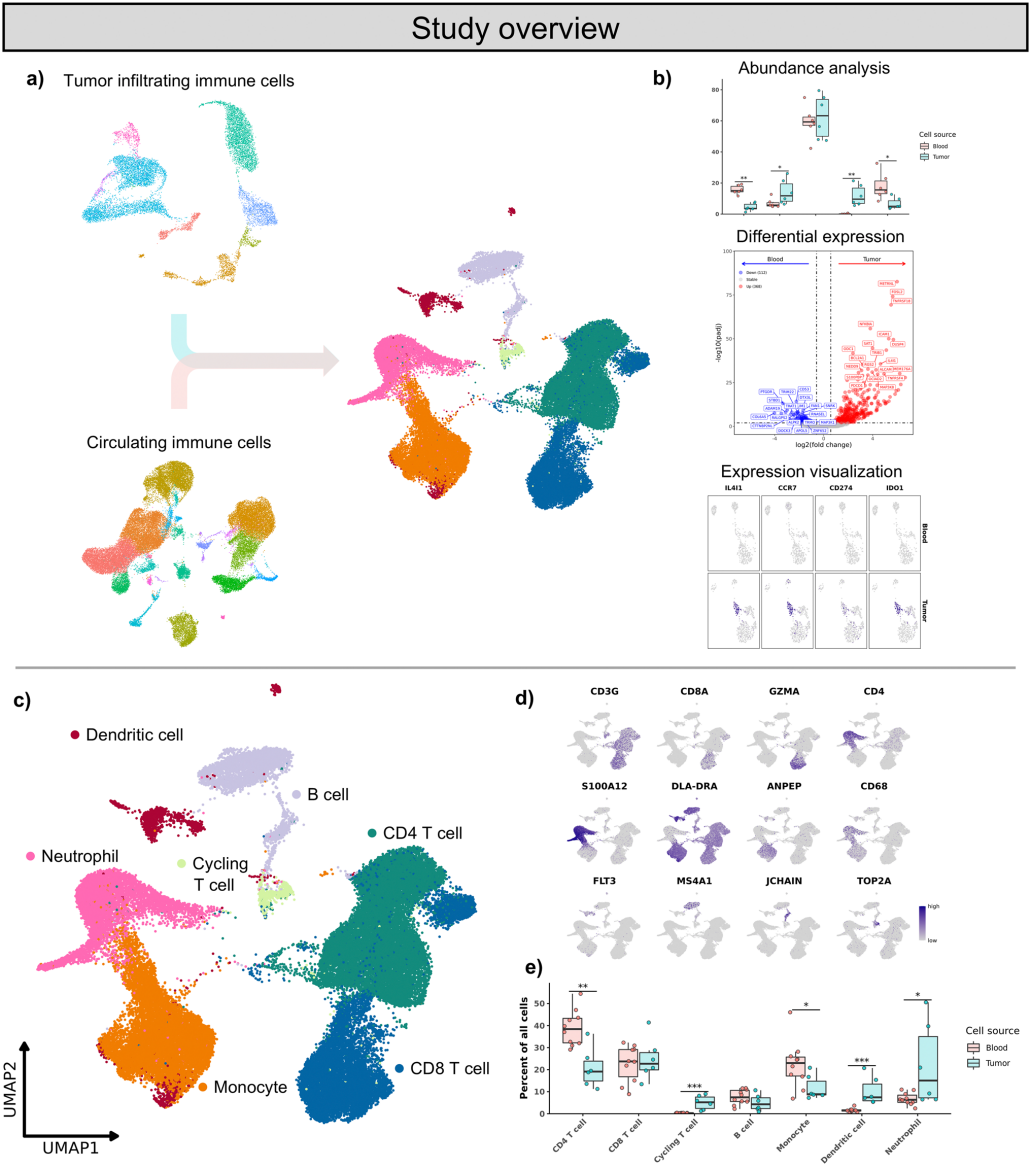
Study overview and cell type annotations. **a**/**b** Diagram of study design. Tumor-infiltrating immune cell and circulating immune cell were integrated into one dataset then transcriptomic and cell type abundances differences were evaluated. **c** UMAP representation with major cell type annotations of integrated circulating immune cells from 10 dogs with osteosarcoma (n = 42,292 cells) and tumor-infiltrating immune cells from 6 treatment-naïve OS dogs (n = 11,431 cells). **d** Feature plots depicting canonical cell type markers. **e** Boxplots depicting distribution of cell type percentages within blood and tumor. Significance determined using two-sided Wilcoxon rank sum test. *** = *P* value < 0.001; ** = *P* value < 0.01; * = *P* value < 0.05

The canine datasets used in this study consisted of 10 blood leukocyte samples (n = 37,887 cells) from dogs with primary OS and tumor-infiltrating immune cells from 6 treatment-naïve dogs with primary OS of the axial skeleton (n = 11,257 cells) (Fig. 1a, b, Supplemental Table 5). Analysis of the OS-associated impacts on immune cells focused on six major cell types: neutrophils, monocytes, CD4 T cells, CD8 T cells, B cells, and dendritic cells (DCs), plus a minor cluster of cycling T cells (Fig. 1c, d). Evaluation of relative abundance shifts in the OS TME versus blood revealed a greater proportion of CD4 T cells and monocytes in blood and relative proportion increases of DCs and neutrophils within tumors (Fig. 1e). Additionally, we found cycling T cells exhibited a marked increase in the relative proportion of cells within the tumor samples (4.52% ± 2.97) relative to blood samples (0.29% ± 0.25) (Supplemental Table 6). We then further subset each major cell type and conducted a detailed comparison between infiltrating and circulating cells.

### Follicular helper and regulatory CD4 T cells are overrepresented in the tumor microenvironment

**Fig. 2:**
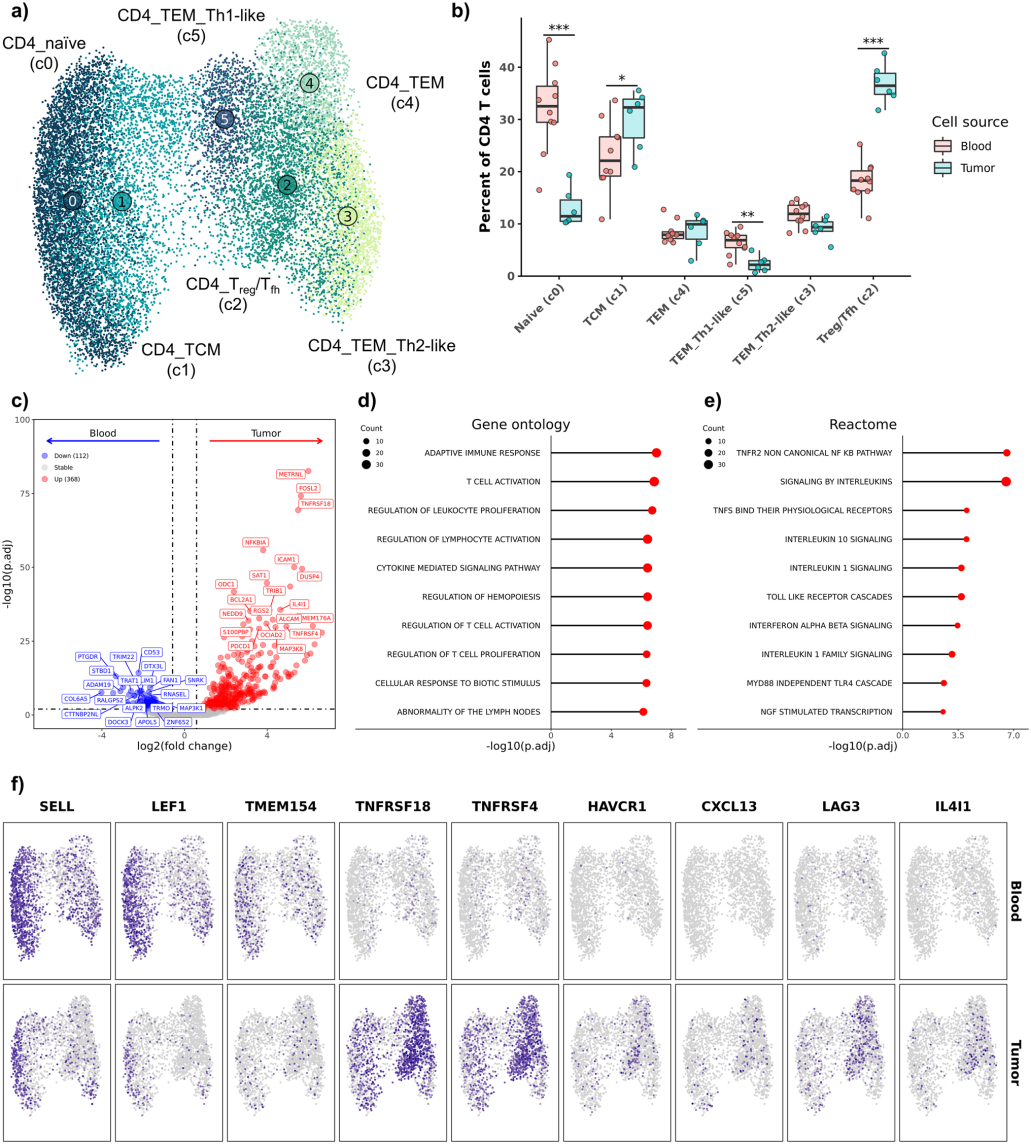
Tumor-infiltrating cells exhibit higher relative proportions of follicular helper and regulatory CD4 T cell relative to circulating CD4 T cells. **a** UMAP representation of 16,765 CD4 T cells colorized by cell type annotation. **b** Boxplots depicting distribution of cell type percentages within blood and tumor. Significance determined using two-sided Wilcoxon rank sum test. *** = *P* value < 0.001; ** = *P* value < 0.01; * = *P* value < 0.05. **c** Volcano plot depicting results of differential gene expression analysis contrasting all tumor-infiltrating CD4 T cells versus all circulating CD4 T cells. Lollipop charts depicting log10 transformed adjusted P values from gene set enrichment analysis using genes upregulated in tumor-infiltrating CD4 T cells against gene ontology (**d**) and Reactome (**e**) gene sets. **f** Feature plots split by tissue source depicting normalized expression of differentially expressed features

After transferring cell type annotations to the CD4 T cell subset, we identified 6 distinct CD4^+^ T cell populations in the combined tumor-blood dataset. The cell types consisted of naïve, central memory (TCM), effector memory (TEM), Th1-like TEM, Th2-like TEM, and regulatory/follicular helper T cells (*T*_reg_/*T*_fh_) (Fig. 2a, Supplemental Fig. 2a, Supplemental Data 1). The cluster annotated as *T*_reg_/*T*_fh_ did not reach a consensus when transferring cell type labels and was annotated based on the presence of both regulatory and follicular helper T cells (Supplemental Fig. 2b). Of the six CD4 T cell populations identified, naïve T cells and Th1-like TEM cells were found to be more abundant in blood compared to the TME, while TCM CD4 T cells and *T*_reg_/*T*_fh_ cells were overrepresented in the tumor (Fig. 2b, Supplemental Table 7). To further investigate how heterogeneity within the *T*_reg_/*T*_fh_ cluster impacted differential abundance analysis, we used CXCL13 expression (a molecule essential for *T*_fh_ mediated B cell recruitment) as a proxy for *T*_fh_ cells to better understand *T*_fh_ presence in the TME [24, 25]. Through evaluation of CXCL13^+^ cells (normalized count > 0) within the *T*_reg_/*T*_fh_ cluster we found that CXCL13^+^ *T*_fh_ were almost exclusively found in TME (Blood: 2/14583; Tumor: 121/2182 CXCL13^+^ cells) (Supplemental Fig. 2c). This suggests that the overrepresentation of *T*_reg_/*T*_fh_ CD4 T cells in the tumor, was in part due to the increased proportion of CXCL13^+^ *T*_fh_ cells.

Next, we completed DE analysis within CD4 T cells to compare expression profiles between tumor-infiltrating lymphocytes (TILs) and circulating lymphocytes. The analysis revealed 368 genes to be more highly expressed in CD4 TILs and 112 genes to be overexpressed in CD4 blood leukocytes (Fig. 2c, Supplemental Data 2). Subsequent GSEA using the genes enriched in CD4 TILs revealed enrichment for multiple terms associated with leukocyte activation, proliferation, interleukin signaling and interferon responses (Fig. 2d, e, Supplemental Data 3). Completion of DE analysis and GSEA within each T cell cluster identified that naïve T cells had the strongest enrichment of terms associated with T cell activation and differentiation, while TCM and *T*_reg_/*T*_fh_ had the strongest enrichment of terms associated with regulation of immune responses (Supplemental Fig. 3a). Select DEGs were then visualized to identify which clusters were driving differential expression (Fig. 2f, Supplemental Fig. 3b, c). The expression patterns revealed that overexpression of SELL and LEF1 in blood leukocytes was associated with naïve T cells, while exhaustion (HAVCR1, PDCD1, LAG3) and activation (TNFRSF4, TNFRSF18) markers overexpressed in CD4 TILs were largely confined to TEM and *T*_reg_/*T*_fh_ cell types (Supplemental Fig. 2d) [8, 9]. Consistent with reports in human melanoma and head and neck cancer [26, 27], our analysis suggests that CD4 TILs exhibit altered transcriptional profiles suggestive of activation and exhaustion relative to their circulating CD4 T cell counterparts.

### Features associated with T cell exhaustion are enriched in tumor-infiltrating effector CD8 T cells

**Fig. 3:**
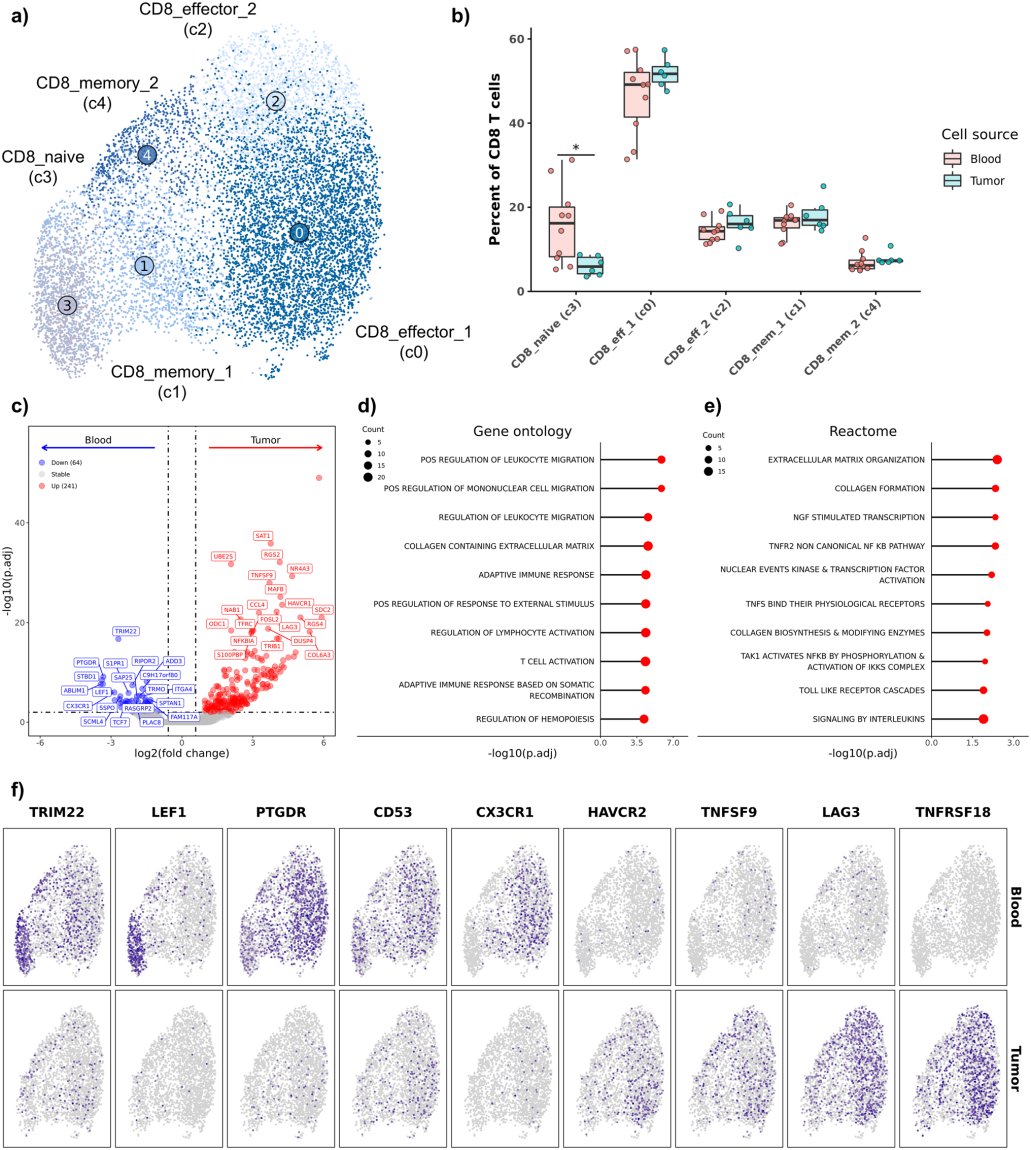
Tumor-infiltrating CD8 T cells overexpress T cell exhaustion markers. **a** UMAP representation of 10,962 CD8 T cells colorized by cell type annotation. **b** Boxplots depicting distribution of cell type percentages within blood and tumor. Significance determined using two-sided Wilcoxon rank sum test. *** = *P* value < 0.001; ** = *P* value < 0.01; * = *P* value < 0.05. **c** Volcano plot depicting results of differential gene expression analysis contrasting all tumor-infiltrating CD8 T cells versus all circulating CD8 T cells. Lollipop charts depicting -log10 transformed adjusted *P* value from gene set enrichment analysis using genes upregulated in tumor-infiltrating CD8 T cells against gene ontology (**d**) and Reactome (**e**) gene sets. **f** Feature plots split by tissue source depicting normalized expression of differentially expressed features

Unsupervised clustering of CD8 T cells and NK cells identified 5 transcriptomically distinct clusters which largely matched with the original cell type annotations (Fig. 3a, Supplemental Fig. 4a, Supplemental Data 4). One cell type, NK cells, could not be resolved as a distinct cluster in the integrated dataset. We found that previously annotated NK cells were interspersed within CD8 effector T cell clusters suggesting a substantial overlap in the gene signatures of CD8 T cells and NK cells (Supplemental Fig. 4b). Differential abundance analysis revealed that naïve CD8 T cells were the only subcluster to exhibit an overrepresentation in blood (Fig. 3b, Supplemental Table 8).

When comparing gene expression profiles between CD8 TILs and circulating CD8 T cells, we identified 64 features to be more highly expressed in blood and 241 more highly expressed in the CD8 TILs. (Fig. 3c, Supplemental Data 2). Multiple T cell exhaustion markers including LAG3, TNFSF9 (4-1BB ligand), and HAVCR2 (TIM-3) [8], were identified to be more abundantly expressed in CD8 TILs. Completion of enrichment scoring using an exhaustion gene signature further supported significant increases in expression of exhaustion genes across all CD8 T cell subsets (Supplemental Fig. 4c). Despite filtering to exclude features associated with background tissue gene signatures, GSEA indicated that CD8 TIL gene expression profiles were associated with extracellular matrix processes (Fig. 3d, Supplemental Data 3). This enrichment pattern could be an artifact driven by tissue signatures or it is possible that the results represent expression changes associated with the transition from circulating to tissue infiltrating T cells.

The GSEA results further revealed CD8 TILs to be associated with T cell activation and recruitment of mononuclear cells. Reactome analysis identified multiple terms associated with NF-κB signaling to be enriched in CD8 TILs, which suggests increased T cell activation (Fig. 3e, Supplemental Data 3) [28]. Visualization of DEGs revealed that the increased expression of LEF1 in blood leukocytes was associated with naïve CD8 T cells, while the increased CX3CR1/PTGDR expression was associated with effector CD8 T cells (Fig. 3f, Supplemental Fig. 5). Tumor-infiltrating effector CD8 T cells were identified as drivers of LAG3, TNFSF9, and HAVCR2 overexpression, which suggests effector CD8 TILs are activated and exhausted relative to circulating leukocytes, a finding that is consistent with reports across multiple human tumor types [8, 9].

### Tumor-infiltrating B cells upregulate FOS and have gene expression patterns suggestive of activation

**Fig. 4:**
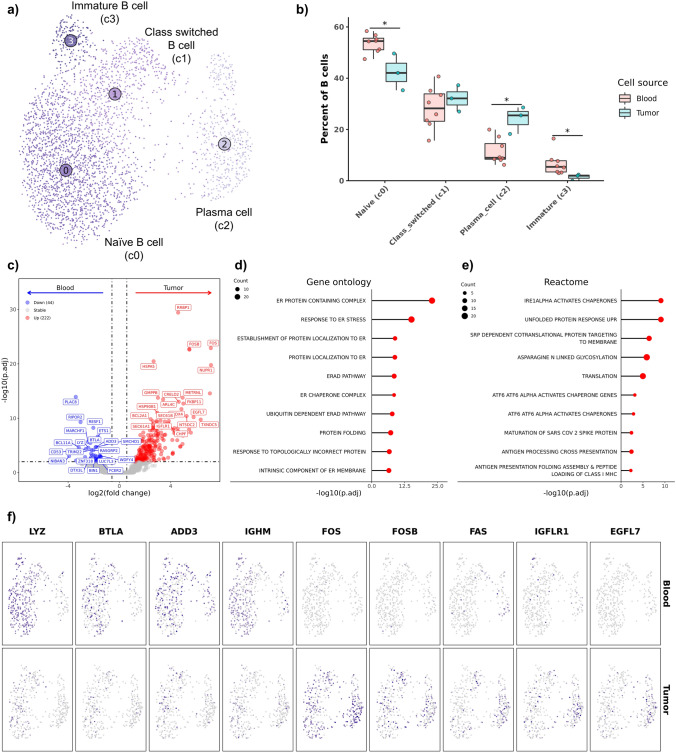
Tumor-infiltrating B cells exhibit gene expression patterns suggestive of antigen presentation and maturation. **a** UMAP representation of unsupervised clustering of 3,108 B cells. **b** Boxplots depicting distribution of cell type percentages within blood and tumor. Significance determined using two-sided Wilcoxon rank sum test. *** = *P* value < 0.001; ** = *P* value < 0.01; * = *P* value < 0.05. **c** Volcano plot depicting results of differential gene expression analysis contrasting all tumor-infiltrating B cells versus all circulating B cells. Lollipop charts depicting -log10 transformed adjusted *P* values from gene set enrichment analysis using genes upregulated in tumor-infiltrating B cells against gene ontology (**d**) and Reactome (**e**) gene sets. **f** Feature plots split by tissue source depicting normalized expression of differentially expressed features

Integration of circulating and tumor-infiltrating B cells revealed the presence of 3 B cell subtypes (immature, naïve, and class switched) and a cluster of plasma cells (Fig. 4a, Supplemental Fig. 6a, Supplemental Data 5). Differential abundance analysis indicated an increase in the relative proportion of immature B cells and naïve B cells in blood, while an increase in the relative proportion of plasma cells was identified within the TME (Fig. 4b, Supplemental Table 9). Through evaluation of Euclidean distance of each cluster, we identified plasma cells to be distantly related to the B cell subtypes, suggesting plasma cells should be treated as a distinct cell type (Supplemental Fig. 6b). As such, we completed DE analysis within plasma cells separately from the B cell subsets. DE analysis within the plasma cells revealed relatively few differentially expressed genes (27 features over expressed in tumor-infiltrating plasma cells and 15 features over expressed on circulating plasma cells), implying only subtle tumor-associated changes in gene expression (Supplemental Fig. 6c). Completion of DE analysis within in B cell subsets (c0, c1, and c3) between tissue sources revealed 44 features to be more highly expressed in circulating B cells and 222 features to be more highly expressed in infiltrating B cells (Fig. 4c, Supplemental Data 2). Top DGEs included the proto-oncogenes, FOS and FOSB, which were identified as two of the most upregulated features in tumor-infiltrating B cells. The expression of FOS family gene members in B cells has been associated with terminal differentiation following interaction with a cognate antigen [29], but has also been associated with activation of apoptotic pathways in B cells [30].

To investigate gene expression patterns in B cells further, we utilized GSEA which revealed enrichment of several terms associated with endoplasmic reticulum-associated degradation (ERAD), protein modification, endoplasmic reticulum activity, and antigen presentation in tumor-infiltrating B cells (Fig. 4d, Supplemental Data 3). The enrichment of ERAD and endoplasmic reticulum stress pathways further suggested that B cells within tumor tissues may be undergoing apoptosis through ER stress-induced cell death [31]. It is also possible that these pathways were enriched due to increased antigen processing and presentation, as this has been supported in human pan-cancer and breast cancer scRNA-seq studies [32, 33]. The possible role in antigen presentation was further supported by Reactome enrichment analysis which identified enrichment of multiple terms associated with antigen presentation (3/15 enriched Reactome terms were related to antigen presentation) (Fig. 4e). Additional investigation is needed to determine the functional implication of the transcriptomic changes observed within the tumor-infiltrating B cells. Visualization of select DEGs, indicated that IGHM expression was broadly reduced in tumor-infiltrating B cells, suggesting differentiation away from a naïve gene signature (Fig. 4f, Supplemental Fig. 6d) [34]. Overall, these findings indicate that tumor-infiltrating B cells become activated upon entry into the OS TME and that B cells may participate in tumor antigen processing and presentation.

### Mature regulatory dendritic cells (mregDCs) are abundant in the OS tumor microenvironment, but rare in circulation

**Fig. 5:**
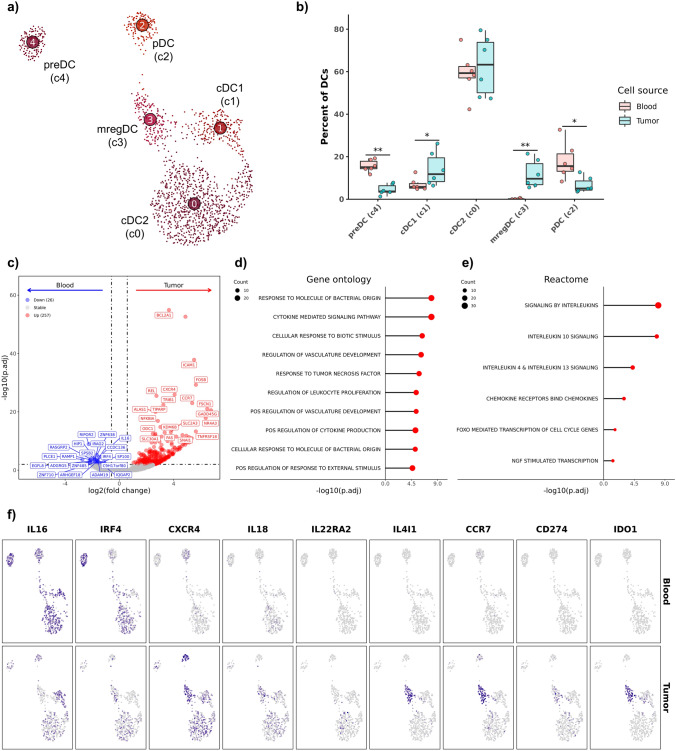
Mature regulatory dendritic cells are abundant in OS tumors, but not in circulation. **a** UMAP representation of unsupervised clustering of 1,556 dendritic cells. **b** Boxplots depicting distribution of cell type percentages within blood and tumor. Significance determined using two-sided Wilcoxon rank sum test. *** = *P* value < 0.001; ** = *P* value < 0.01; * = *P* value < 0.05. **c** Volcano plot depicting results of differential gene expression analysis contrasting all tumor-infiltrating dendritic cells versus all circulating dendritic cells. Lollipop charts depicting -log10 transformed adjusted P values from gene set enrichment analysis using genes upregulated in tumor-infiltrating dendritic cells against gene ontology (**d**) and Reactome (**e**) gene sets. **f** Feature plots split by tissue source depicting normalized expression of differentially expressed features

Five DC subtypes were identified in the integrated dataset (Fig. 5a, Supplemental Fig. 7a, Supplemental Data 6). Of the subtypes, we identified plasmacytoid DCs (pDCs) and precursor (pre) DCs to be overrepresented in blood relative to the TME (Fig. 5b, Supplemental Table 10). In contrast, mature regulatory DCs (mregDCs), a recently described immune modulatory population [6], were the only DC subpopulation to be classified as unique to the OS TME (Blood: 0.11% ± 0.26; Tumor: 11.31% ± 6.69). Lastly, conventional type 1 DCs (cDC1s) were determined to have an increased abundance in the OS TME realtive to blood. The identification of mregDCs as unique to the TME has been previously documented in human cancers [35, 36]. Although we did not investigate how mregDCs accumulated in the canine OS TME, the marked overrepresentation suggests a conserved role of mregDCs between the two species.

To investigate how the TME impacted DC gene expression, we completed DE analysis and subsequent GSEA. The analysis revealed 257 features enriched in tumor-infiltrating DCs and 26 enriched in circulating DCs (Fig. 5c, Supplemental Data 2). Gene ontology analysis revealed associations with tumor necrosis factor (TNF) responses and vascular development within tumor-infiltrating DC (Fig. 5d, Supplemental Data 3). GSEA using Reactome terms identified increased interleukin activity of tumor-infiltrating DCs with IL4, IL13, and IL10 predicted to elicit the greatest impact on infiltrating DCs (Fig. 5e, Supplemental Data 3). Further analysis using ImmuneSigDB indicated tumor DCs are activated following infiltration (Supplemental Fig. 7b). The relative expansion of mregDCs impacted DE analysis (as evidenced by CCR7, IDO1, IL4I1, and CD274 upregulation), so we further investigated which cell types were associated with DEGs. We identified IL16 and IRF4 to be broadly downregulated in tumor-infiltrating DCs, CXCR4 to be broadly upregulated, and IL18 and IL22RA2 increases to be associated with cDC2s (Fig. 5f, Supplemental Fig. 7c). In summary, we provide evidence that canine mregDCs are abundant in the OS TME and that infiltrating DCs are predicted to participate in interleukin signaling.

### Tumor-infiltrating monocytes upregulate chemokine and immunoregulatory molecule expression relative to circulating monocytes

**Fig. 6:**
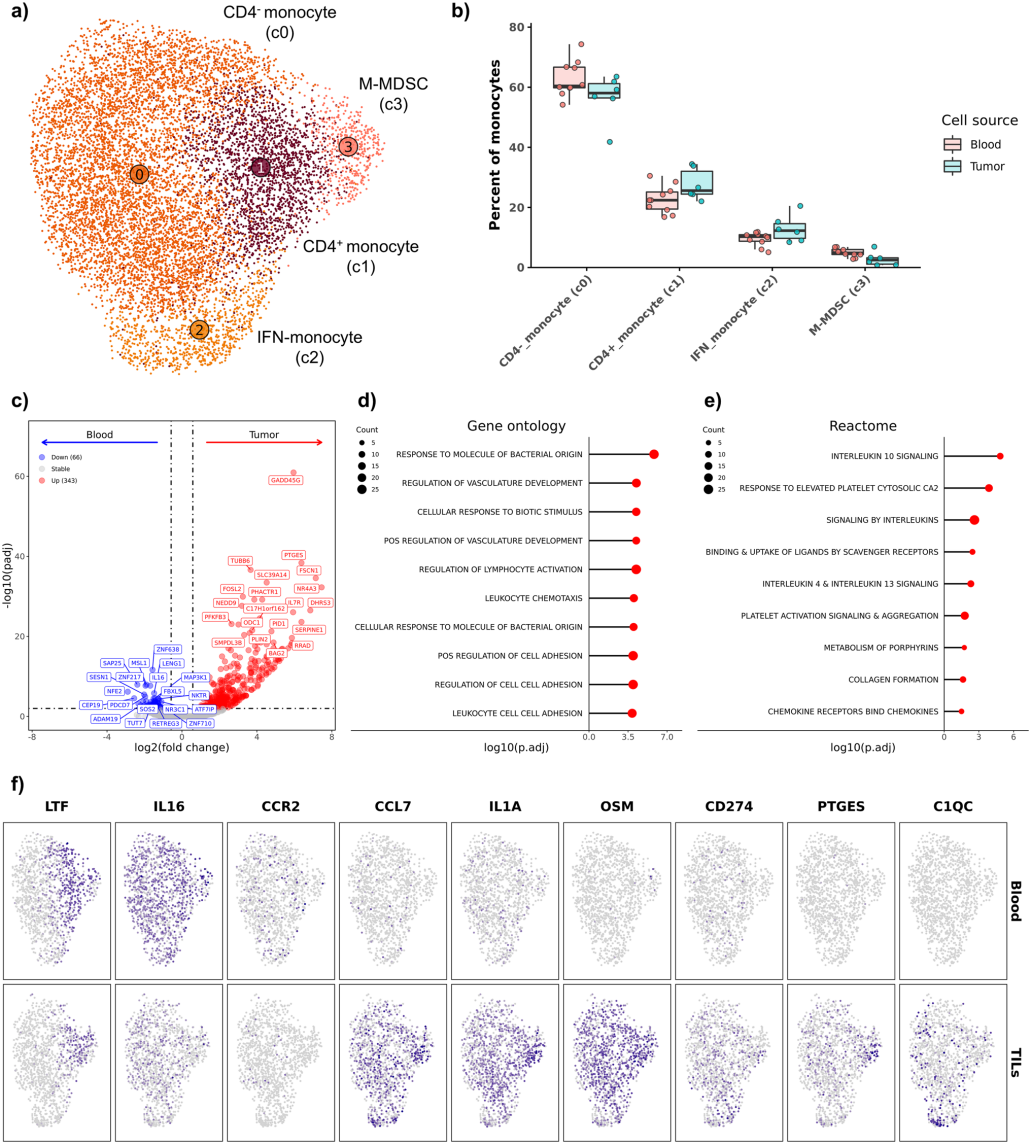
Tumor-infiltrating monocytes upregulate inflammatory and immune modulatory molecules relative to circulating monocytes. **a** UMAP representation of unsupervised clustering of 10,004 monocytes. **b** Boxplots depicting distribution of cell type percentages within blood and tumor. Significance determined using two-sided Wilcoxon rank sum test. *** = *P* value < 0.001; ** = *P* value < 0.01; * = *P* value < 0.05. **c** Volcano plot depicting results of differential gene expression analysis contrasting all tumor-infiltrating monocytes versus all circulating monocytes. Lollipop charts depicting -log10 transformed adjusted P values from gene set enrichment analysis using genes upregulated in tumor-infiltrating monocytes against gene ontology (**d**) and Reactome (**e**) gene sets. **f** Feature plots split by tissue source depicting normalized expression of differentially expressed features

Evaluation of cell type abundance shifts within tumor-infiltrating and circulating monocytes revealed no significant differences in the distribution of the four monocyte subtypes (Fig. 6a, b, Supplemental Data 7, Supplemental Table 11). Despite a lack of changes in relative monocyte abundances, DE analysis revealed marked transcriptomic changes induced by tumor infiltration with 356 features enriched in tumor-infiltrating monocytes (TIMs) and 69 enriched in blood monocytes (Fig. 6c, Supplemental Data 2). Pathway analysis of the features identified to be overexpressed in TIMs revealed enrichment of terms associated with general immune activation, adhesion, and interleukin signaling. (Fig. 6d, e, Supplemental Data 3). Given the non-specificity of the enriched terms, we use ImmuneSigDB to provide further functional annotation of the DEGs. The analysis showed enrichment of lipopolysaccharide and interferon treated monocytes, indicating that TIMs exhibit an inflammatory monocyte gene signature relative to circulating monocytes (Supplemental Fig. 8a).

Visualization of DEGs demonstrated IL16 and FGL2 (Fibrinogen-like protein 2) to be broadly expressed features; whereas, LTF (lactotransferin) expression was primarily localized to myeloid-derived suppressor cells (M-MDSCs) (Fig. 6f, Supplemental Fig. 9a–c). Further investigation of DEG expression patterns identified TIMs to generally exhibit increased expression of multiple chemokines (CXCL10, CXCL16, CCL19, CCL5, CCL7, CCL8) (Supplemental Fig. 9d). Relative to blood monocytes, immune modulatory molecules (IL1A, OSM, CD274, and PTGES) were determined to be broadly upregulated in TIMs. Lastly, the overexpression of C1QC in TIMs relative to blood monocytes may represent that cells are transitioning to tumor-associated macrophage (TAM). It is also possible that during data filtering some TAMs were unintentionally included in the analysis and impacted DE analysis.

To further investigate the driver of TAM-associated genes being enriched in TIMs and to assess the relationship between TIMs, TAMs, and blood monocytes, we completed hierarchical clustering of the three cell types. The analysis revealed that TIMs and circulating monocytes clustered together, while TAMs were on a distinct clade (Supplemental Fig. 8b). Furthermore, more DEGs were identified when comparing the expression profiles of TAMs to blood monocytes than comparing TIMs to blood monocytes (Supplemental Fig. 8c). Overall, we found TIMs to be more closely related to blood monocytes than TAMs, identified TIMs to be enriched in inflammatory monocyte gene signatures, and identified an upregulation of immune modulatory molecules in TIMs relative to blood monocytes.

### Tumor-associated neutrophils increase oncostatin M and chemokine expression relative to circulating neutrophils

**Fig. 7:**
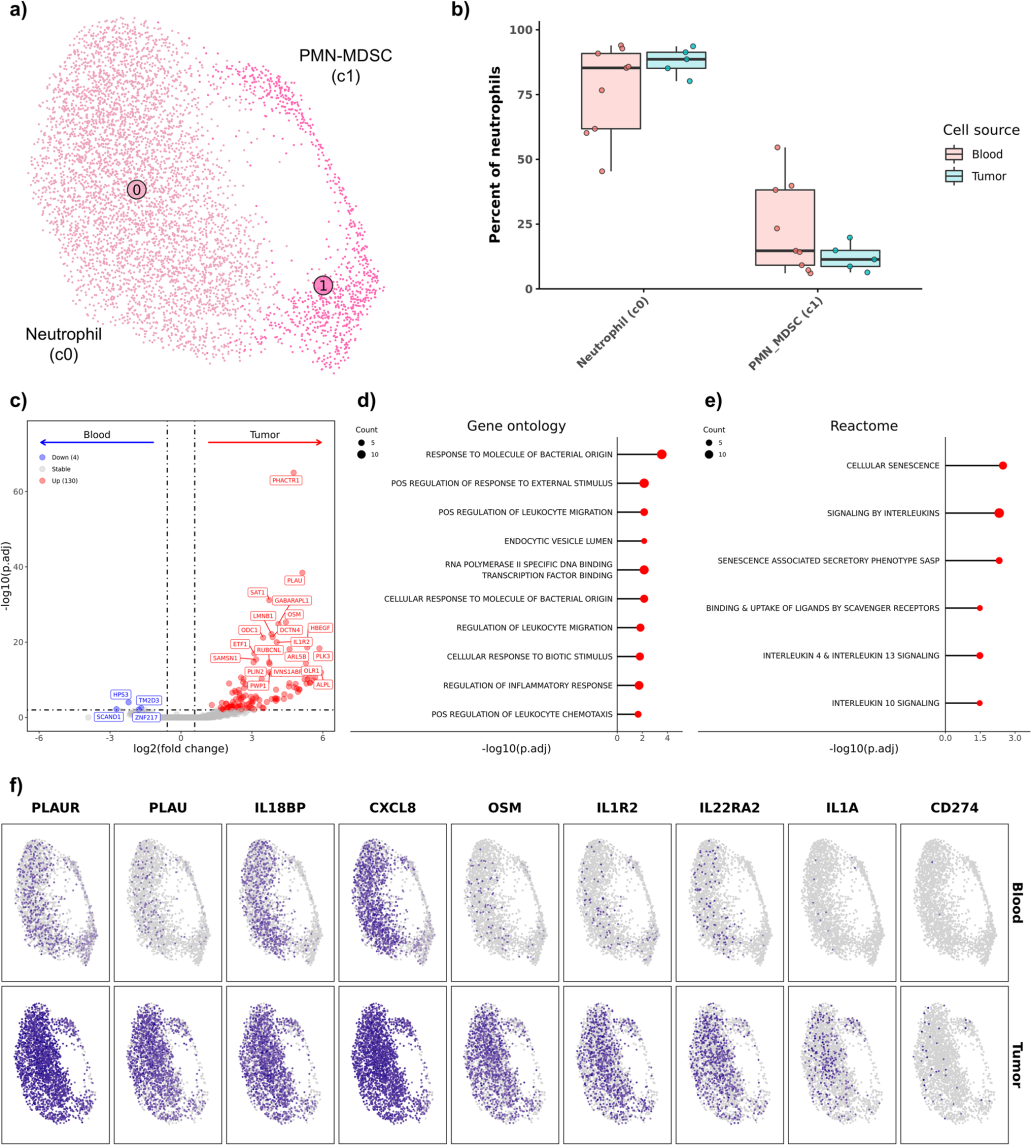
Tumor-infiltrating neutrophils increase expression of activation and inflammatory markers relative to circulating neutrophils. **a** UMAP representation of unsupervised clustering of 5,470 neutrophils. **b** Boxplots depicting distribution of cell type percentages within blood and tumor. Significance determined using two-sided Wilcoxon rank sum test. *** = *P* value < 0.001; ** = *P* value < 0.01; * = *P* value < 0.05. **c** Volcano plot depicting results of differential gene expression analysis contrasting all tumor-infiltrating neutrophils versus all circulating neutrophils. Lollipop charts depicting -log10 transformed adjusted P values from gene set enrichment analysis using genes upregulated in tumor-infiltrating neutrophils against gene ontology (**d**) and Reactome (**e**) gene sets. **f** Feature plots split by tissue source depicting normalized expression of differentially expressed features

Evaluation of tumor-associated neutrophils (TANs) and circulating neutrophils revealed no changes in the relative proportions of neutrophils (c0) and PMN-MDSCs (c1), indicating the ratio of PMN-MDSCs to neutrophils is consistent in the blood and OS TME (Fig. 7a/b, Supplemental Data 8, Supplemental Table 12). DE analysis identified upregulation of 139 features in TANs, with only 4 features preferentially expressed in the blood (Fig. 7c, Supplemental Data 2). Subsequent pathway analysis suggested TAN transcriptomic signatures were associated with general neutrophil activation, responses to interleukins, and response to endoplasmic reticulum stress (Fig. 7d/e, Supplemental Data 3). Together the analysis indicates that TANs exhibited a shift toward an activated state with increased enrichment of gene programs associated with cell migration and interleukin signaling.

Oncostatin M (OSM), a member of the IL-6 family with reported immune suppressive properties, and CD274 (PD-L1) were found to be broadly upregulated in TANs (Fig. 7f, Supplemental Fig. 10a–c) [37]. TANs were also found to broadly overexpress multiple chemokines (CCL5, CCL7, and CXCL8), which suggests that upon infiltration TANs secrete chemokines that have the potential to promote myeloid cell infiltration (Supplemental Fig. 10d). Plasminogen activator urokinase and its receptor (PLAU and PLAUR), were also enriched in TANs. Both PLAU and PLAUR have been associated with neutrophil activation and infiltration, suggesting PLAU-PLAUR interactions may be important for neutrophil infiltration into canine OS [38]. IL1R2, a decoy receptor for IL1A/B, was broadly upregulated in TANs which suggests TANs may function to dampen inflammatory responses [39]. Our analysis was consistent with similar analysis completed in human non-small cell lung cancer in which PLAU, PLAUR, IL1R2, CD274, OSM, and CXCL8 were reported to be significantly enriched in TANs relative to circulating neutrophils [40]. Overall, our findings indicate that TANs broadly upregulated immune suppressive molecules upon infiltration and suggest that TANs play a role in shaping the immune suppressive TME.

### Human–canine comparative transcriptomic analysis reveals conserved and divergent responses to tumor infiltration between species

**Fig. 8:**
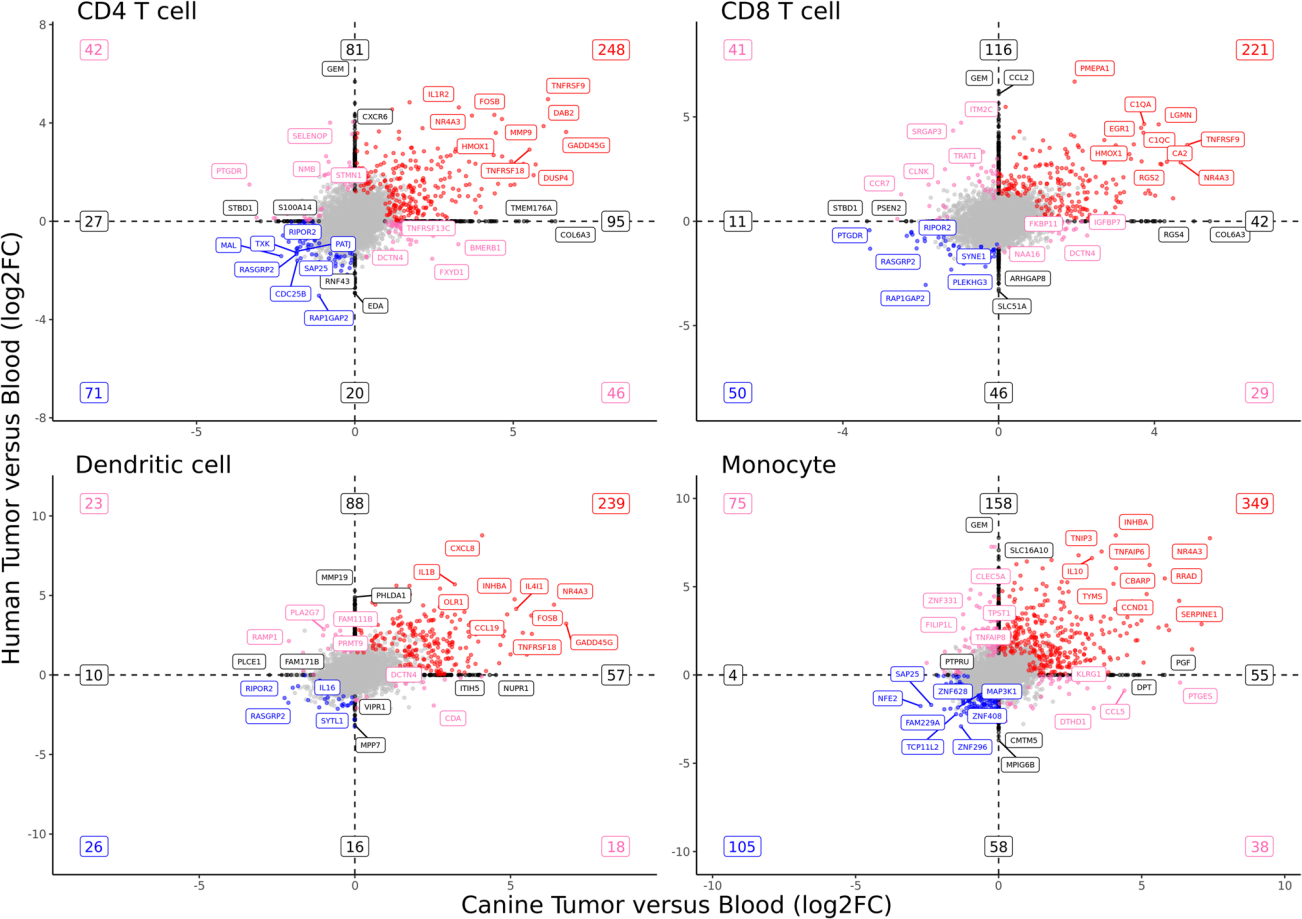
Characterization of conserved and divergent responses to tumor infiltration through comparative human–canine transcriptomic analysis. Scatter plots depicting the log2(Fold change) for every gene evaluated when contrasting human tumor-infiltrating cells versus circulating (y-axis) and canine tumor-infiltrating cells versus circulating (x-axis) for CD4 T cells, CD8 T cells, dendritic cells, and monocytes. Conserved upregulated features associated with tumor-infiltrating are in the top right quadrant (red labels) and conserved downregulated features (enriched in circulating immune cells) are in the bottom left quadrant (blue labels). Conflicting features—up in human but down in dog (bottom right quadrant) and down in human but up in dog (top left quadrant)—are labeled in pink. Features up or down in one species, but not expressed in the other species, are depicted in black on the axis. Features that were not significantly differentially expressed in either species are gray. The numbers in the corners and at the ends of axis lines represent how many features fell in that region

Following characterization of canine transcriptomic responses within major cell types, we next sought to use human OS and blood leukocyte data to compare tumor infiltration transcriptomic signatures between species (Fig. 8, Supplemental Data 9). Of genes identified to be DE in at least one species, approximately 50% of genes (CD4: 51%, CD8: 49%, DC: 56%, Monocyte: 54%) were identified to be conserved between species. Whereas approximately 10% of genes (CD4: 14%, CD8: 13%, DC: 9%, Monocyte: 13%) within each major subset were identified to have fold changes with the opposite direction of change, suggesting a degree of divergent expression patterns between humans and dogs. The remaining approximately 40% of genes (CD4: 35%, CD8: 39%, DC: 36%, Monocyte: 33%) were DE in one species, but not expressed within the major immune cell type being evaluated in the other species. While these genes are divergent in nature, the lack of expression in one species may be more indicative of differences in cell type gene signatures, rather than distinct transcriptomic responses to tumor infiltration.

GSEA of canine and human DEGs using IMMUNESigDB revealed greater than 60% overlap (CD4: 670/899, CD8: 77/121, DC: 281/404, Monocyte: 187/250) between canine and human enriched pathways, suggesting conserved transcriptomic responses to tumor infiltration (Supplemental Fig. 11, Supplemental Data 10). Although the most strongly enriched pathways consistently overlapped between species, analysis identified multiple pathways that were weakly enriched in one species but not the other, indicating a degree of divergence in expression patterns between species. To highlight a few differences between species, we identified PTGES2 and CCL5 expression to be increased in canine TIMs, but not in human TIMs, while the opposite was true for CCR2. This finding indicates that PTGES2 upregulation in TIMs may play a more active role in shaping the canine OS TME compared to the human OS TME [41]. Overall, our cross-species analysis revealed conserved transcriptomic responses to tumor infiltration, but also identified subtle gene expression differences which could translate to functional variation between species.

## Discussion

Immune suppression and evasion are hallmarks of cancer and understanding how immune cells are impacted by the TME is foundational for the development of effective immunotherapies. In the present study we investigated how the canine OS TME modulates the transcriptomic signatures and relative cell type abundance of tumor-infiltrating immune cell. Using circulating leukocytes as a point of reference, we identified upregulated expression of exhaustion markers on tumor-infiltrating T cells and found that mregDCs were abundant in the OS TME, but rarely detected in circulation. Our analysis of TIMs revealed enrichment of inflammatory gene signatures and that TIMs more closely resemble circulating monocytes than TAMs. Furthermore, Oncostatin M (OSM) was found to be more highly expressed on TIMs and TANs relative to their circulating counterparts, implicating these myeloid cell populations as the primary producers of this immune regulatory cytokine [37]. Lastly, our side-by-side analysis using publicly available human data revealed human and canine tumor infiltration transcriptomic responses to be largely conserved, but the analysis also identified genes that exhibited divergent expression patterns. Overall, the findings presented here suggest that the features of tumor-induced immune suppression and exhaustion that have been reported across species are active in canine OS.

Through our analysis we identified upregulation of immune suppressive transcripts in tumor-infiltrating monocytes, neutrophils, and dendritic cells. Immune suppressive roles of myeloid cells within tumors have been described in a large body of literature, and our analysis provides unique insights into which features are upregulated following infiltration into the canine OS TME [42–44]. In particular, our analysis revealed increased expression of various immune modulatory molecules (CD274 (PD-L1), OSM, PTGES2, CD36, and MSR1) by tumor-infiltrating monocytes and neutrophils suggesting an immune suppressive role is adopted following infiltration into the TME. Additionally, we identified IL1R2, a decoy receptor for IL1A/B, to be upregulated in TANs which suggests immunological checkpoint blockage with IL1R2 antagonists may be of therapeutic interest in canine OS [45, 46]. Lastly, our analysis provides evidence that canine mregDCs are unique to the TME relative to peripheral blood, but it is possible that mregDCs are also abundant in other tissues, such as local tumor draining lymph nodes [6, 47].

Consistent with scRNA-seq data investigating tumor-infiltrating T cells across multiple human tumor types [8, 9], we identified increased relative proportions of regulatory and exhausted T cells within the canine OS TME. Our analysis further identified that the relative abundances of naïve and Th1-like TEM T cells were reduced in tumor-infiltrating T cells relative to circulating T cell populations. Outside of cell abundance shifts we identified a broad increase in the expression of features associated with exhaustion (CTLA4, LAG3, HAVCR2, PDCD1, TIGIT) across CD8 effector T cells and most non-naïve CD4 T cell populations [8]. As most of these molecules can also be upregulated in activated T cells, and not only exhausted T cells, our analysis cannot fully distinguish between activated and exhausted T cells [48]. Further experimental investigation of the T cell populations is needed to determine the functional status of the cells described in this study.

There is a growing body of literature suggesting intratumoral B cells play a role in antitumoral responses through antigen presentation and by participating in tertiary lymphoid structure (TLS) formation [49, 50]. Our analysis revealed transcriptomic evidence of profound aberrations to tumor-infiltrating B cell protein processing machinery (protein modification, endoplasmic reticulum activity), which could be indicative of B cell dysfunction, antibody production, or antigen presentation. Given that mregDCs, follicular helper T cells, and B cells were all identified in the TME, it is possible that these cell types could have been interacting within TLS of the tumor [51, 52]. Further investigation using spatial transcriptomics is required to determine if mregDCs, B cells, and *T*_fh_ co-localize within the canine OS TME. Overall, our analysis suggests that B cells are modulated by the canine OS TME and may play a role in shaping adaptive immunity.

To complete a comprehensive human–canine comparison of OS TME-associated transcriptomic changes we incorporated human data that was subjected to the same analysis as the canine data. By completing side-by-side analysis we were able to classify genes as conserved (DEG with consistent fold change direction), divergent (DEG with opposite fold change direction), or ambiguous (DEG only expressed in one species). There are many caveats associated with the analysis including: (1) differences in genome annotation quality between human and dog, (2) the human data being generated from independent research groups, (3) human data being generated from different age groups, and (4) different versions of the 10x Genomics platform being used for data generation of human (v3) and canine (v3.1) datasets. Despite these limitations, the gene classifications provided in Supplemental Data 9 act as a resource for comparative immuno-oncology researchers to identify and further investigate potential species differences. Overall, the analysis provides evidence that canine OS recapitulates many components of the human OS immune landscape and identifies divergent gene expression patterns that investigators should consider when studying canine OS.

Although our findings provide insight into how tumor-infiltrating immune cells differ from blood leukocytes in dogs, it is possible that some of the differences in gene expression may be due to the reference population being in circulation rather than cells present in normal bone. Because normal bone has minimal leukocytes present, the use of circulating leukocytes represents the best approach to explore how infiltrating immune cells are modulated by the OS TME. The greatest limitation of using blood as the point of reference is that certain cell types are not found in circulation which precludes a comprehensive analysis, this is especially true for the study of TAMs. Future work incorporating lymph nodes or other non-circulating immune cells may help to more effectively distinguish between tissue- and tumor-associated changes. Additional limitations of this study include a lack of paired samples and a limited number of tumor-infiltrating immune cells (11,257 cells). Although blood and tumor samples were not obtained from the same dogs, the samples included in the study were all processed by the same laboratory and used the same sequencing platform which acts to limit the potential of confounding effects impacting the conclusions.

## Conclusions

The analysis presented here describes the impact that the canine OS TME has on the transcriptomic signatures of infiltrating immune cells. Through our analysis we identified dysregulation of immune modulatory features with marked changes across all cells investigated in this study, including B cells which historically have not been studied extensively in OS. From a comparative immuno-oncology standpoint, the findings enable connections to be made to human literature and provide insights into how the TME alters immune cell transcriptional profiles in spontaneously occurring canine OS. Overall, our analysis sheds light on how the OS TME modulates immune cell transcriptomes and characterizes conserved/divergent expression patterns in humans and canines.

## Supplementary Information

Below is the link to the electronic supplementary material.

Supplementary file1 (CSV 900 KB)

Supplementary file2 (CSV 1302 KB)

Supplementary file3 (CSV 365 KB)

Supplementary file4 (CSV 45 KB)

Supplementary file5 (CSV 77 KB)

Supplementary file6 (CSV 148 KB)

Supplementary file7 (CSV 59 KB)

Supplementary file8 (CSV 40 KB)

Supplementary file9 (XLSX 2075 KB)

Supplementary file10 (CSV 119 KB)

Supplementary file11 (PPTX 20734 KB)

Supplementary file12 (XLSX 26 KB)

Supplementary file13 (CSV 4012 KB)
