# Supplementary material for: Characterization of canine tumor-infiltrating leukocyte transcriptomic signatures reveals conserved expression patterns with human osteosarcoma
Source: Cancer Immunol Immunother. 2025 Feb 11;74(3):105. doi: 10.1007/s00262-025-03950-3 (PMC11813853; doi:10.1007/s00262-025-03950-3)
Supplement: Supplementary file 11 — Supplementary file11 (PPTX 20734 KB) [file 262_2025_3950_MOESM11_ESM.pptx]

## Slide 1
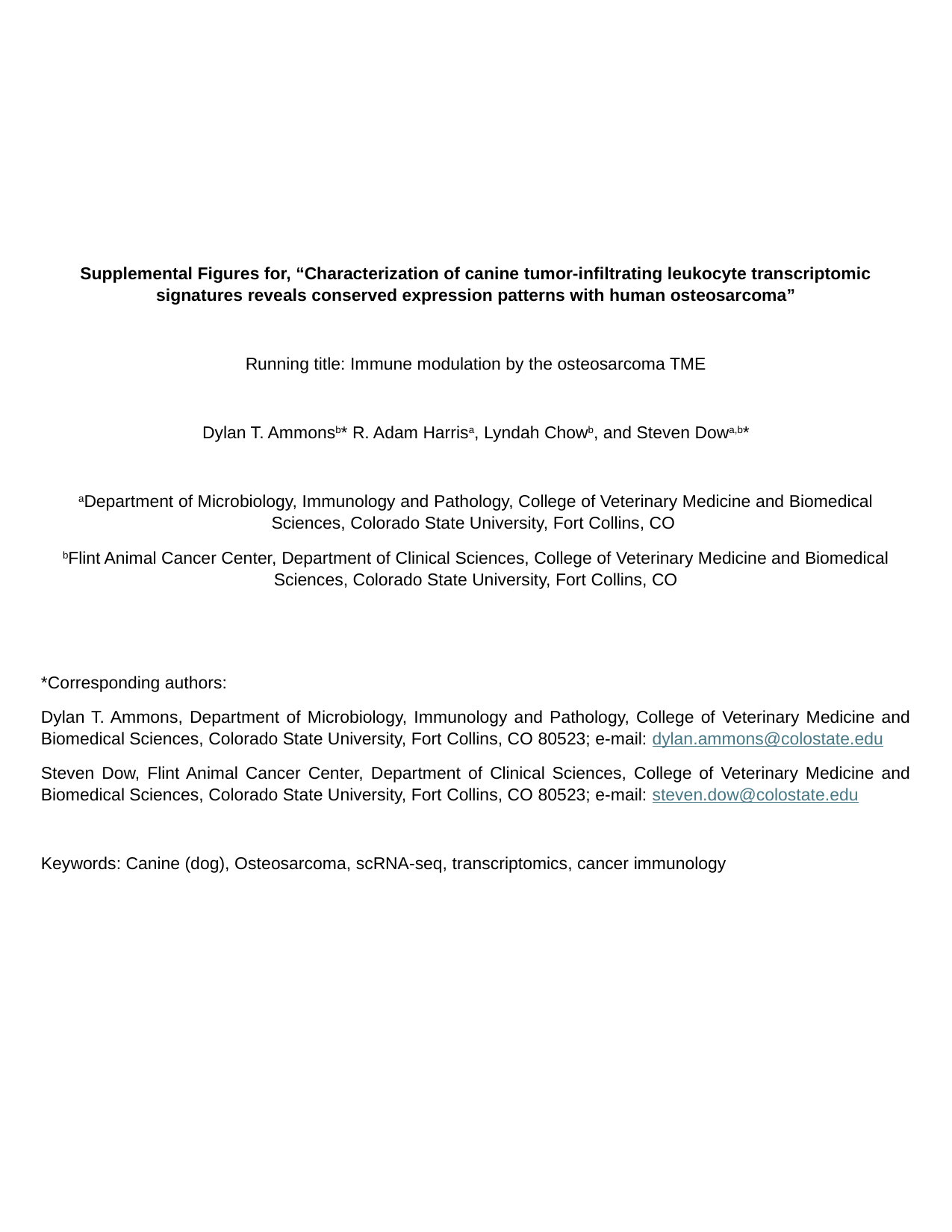

Supplemental Figures for, “Characterization of canine tumor-infiltrating leukocyte transcriptomic signatures reveals conserved expression patterns with human osteosarcoma”
Running title: Immune modulation by the osteosarcoma TME
Dylan T. Ammonsb* R. Adam Harrisa, Lyndah Chowb, and Steven Dowa,b*
aDepartment of Microbiology, Immunology and Pathology, College of Veterinary Medicine and Biomedical Sciences, Colorado State University, Fort Collins, CO
bFlint Animal Cancer Center, Department of Clinical Sciences, College of Veterinary Medicine and Biomedical Sciences, Colorado State University, Fort Collins, CO
*Corresponding authors:
Dylan T. Ammons, Department of Microbiology, Immunology and Pathology, College of Veterinary Medicine and Biomedical Sciences, Colorado State University, Fort Collins, CO 80523; e-mail: dylan.ammons@colostate.edu
Steven Dow, Flint Animal Cancer Center, Department of Clinical Sciences, College of Veterinary Medicine and Biomedical Sciences, Colorado State University, Fort Collins, CO 80523; e-mail: steven.dow@colostate.edu
Keywords: Canine (dog), Osteosarcoma, scRNA-seq, transcriptomics, cancer immunology

## Slide 2
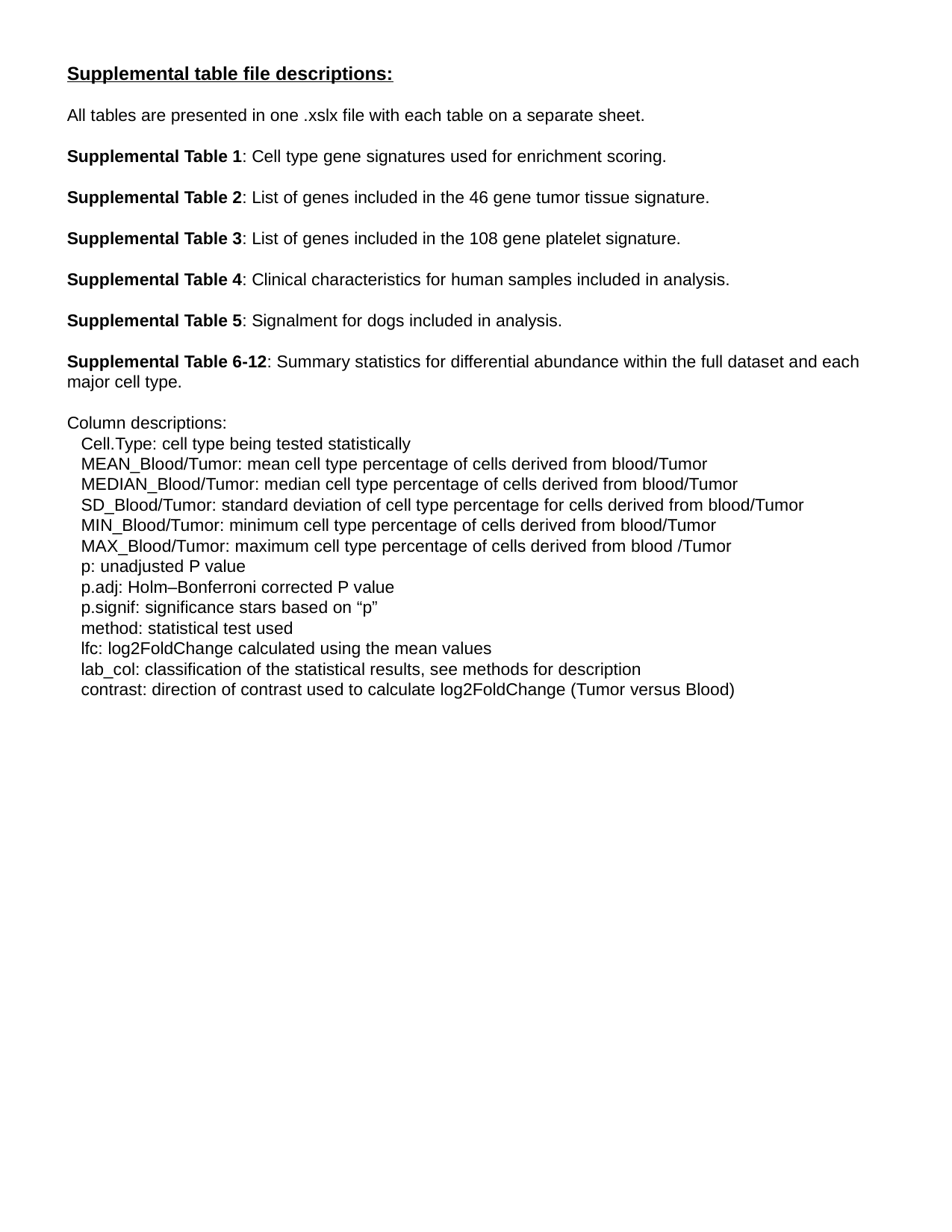

Supplemental table file descriptions:
All tables are presented in one .xslx file with each table on a separate sheet.
Supplemental Table 1: Cell type gene signatures used for enrichment scoring.
Supplemental Table 2: List of genes included in the 46 gene tumor tissue signature.
Supplemental Table 3: List of genes included in the 108 gene platelet signature.
Supplemental Table 4: Clinical characteristics for human samples included in analysis.
Supplemental Table 5: Signalment for dogs included in analysis.
Supplemental Table 6-12: Summary statistics for differential abundance within the full dataset and each major cell type.
Column descriptions:
Cell.Type: cell type being tested statistically
MEAN_Blood/Tumor: mean cell type percentage of cells derived from blood/Tumor
MEDIAN_Blood/Tumor: median cell type percentage of cells derived from blood/Tumor
SD_Blood/Tumor: standard deviation of cell type percentage for cells derived from blood/Tumor
MIN_Blood/Tumor: minimum cell type percentage of cells derived from blood/Tumor
MAX_Blood/Tumor: maximum cell type percentage of cells derived from blood /Tumor
p: unadjusted P value
p.adj: Holm–Bonferroni corrected P value
p.signif: significance stars based on “p”
method: statistical test used
lfc: log2FoldChange calculated using the mean values
lab_col: classification of the statistical results, see methods for description
contrast: direction of contrast used to calculate log2FoldChange (Tumor versus Blood)

## Slide 3
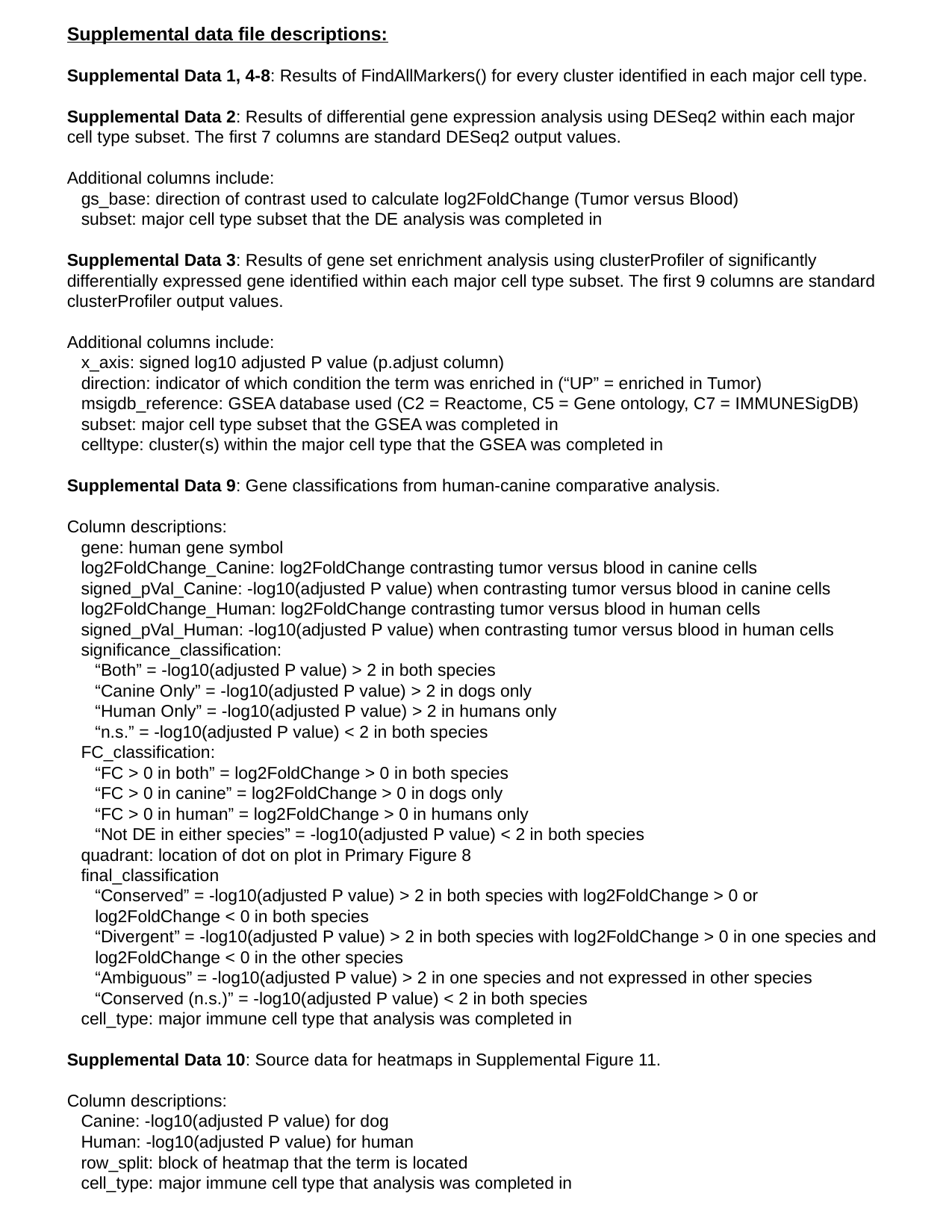

Supplemental data file descriptions:
Supplemental Data 1, 4-8: Results of FindAllMarkers() for every cluster identified in each major cell type.
Supplemental Data 2: Results of differential gene expression analysis using DESeq2 within each major cell type subset. The first 7 columns are standard DESeq2 output values.
Additional columns include:
gs_base: direction of contrast used to calculate log2FoldChange (Tumor versus Blood)
subset: major cell type subset that the DE analysis was completed in
Supplemental Data 3: Results of gene set enrichment analysis using clusterProfiler of significantly differentially expressed gene identified within each major cell type subset. The first 9 columns are standard clusterProfiler output values.
Additional columns include:
x_axis: signed log10 adjusted P value (p.adjust column)
direction: indicator of which condition the term was enriched in (“UP” = enriched in Tumor)
msigdb_reference: GSEA database used (C2 = Reactome, C5 = Gene ontology, C7 = IMMUNESigDB)
subset: major cell type subset that the GSEA was completed in
celltype: cluster(s) within the major cell type that the GSEA was completed in
Supplemental Data 9: Gene classifications from human-canine comparative analysis.
Column descriptions:
gene: human gene symbol
log2FoldChange_Canine: log2FoldChange contrasting tumor versus blood in canine cells
signed_pVal_Canine: -log10(adjusted P value) when contrasting tumor versus blood in canine cells
log2FoldChange_Human: log2FoldChange contrasting tumor versus blood in human cells
signed_pVal_Human: -log10(adjusted P value) when contrasting tumor versus blood in human cells
significance_classification:
“Both” = -log10(adjusted P value) > 2 in both species
“Canine Only” = -log10(adjusted P value) > 2 in dogs only
“Human Only” = -log10(adjusted P value) > 2 in humans only
“n.s.” = -log10(adjusted P value) < 2 in both species
FC_classification:
“FC > 0 in both” = log2FoldChange > 0 in both species
“FC > 0 in canine” = log2FoldChange > 0 in dogs only
“FC > 0 in human” = log2FoldChange > 0 in humans only
“Not DE in either species” = -log10(adjusted P value) < 2 in both species
quadrant: location of dot on plot in Primary Figure 8
final_classification
“Conserved” = -log10(adjusted P value) > 2 in both species with log2FoldChange > 0 or log2FoldChange < 0 in both species
“Divergent” = -log10(adjusted P value) > 2 in both species with log2FoldChange > 0 in one species and log2FoldChange < 0 in the other species
“Ambiguous” = -log10(adjusted P value) > 2 in one species and not expressed in other species
“Conserved (n.s.)” = -log10(adjusted P value) < 2 in both species
cell_type: major immune cell type that analysis was completed in
Supplemental Data 10: Source data for heatmaps in Supplemental Figure 11.
Column descriptions:
Canine: -log10(adjusted P value) for dog
Human: -log10(adjusted P value) for human
row_split: block of heatmap that the term is located
cell_type: major immune cell type that analysis was completed in

## Slide 4
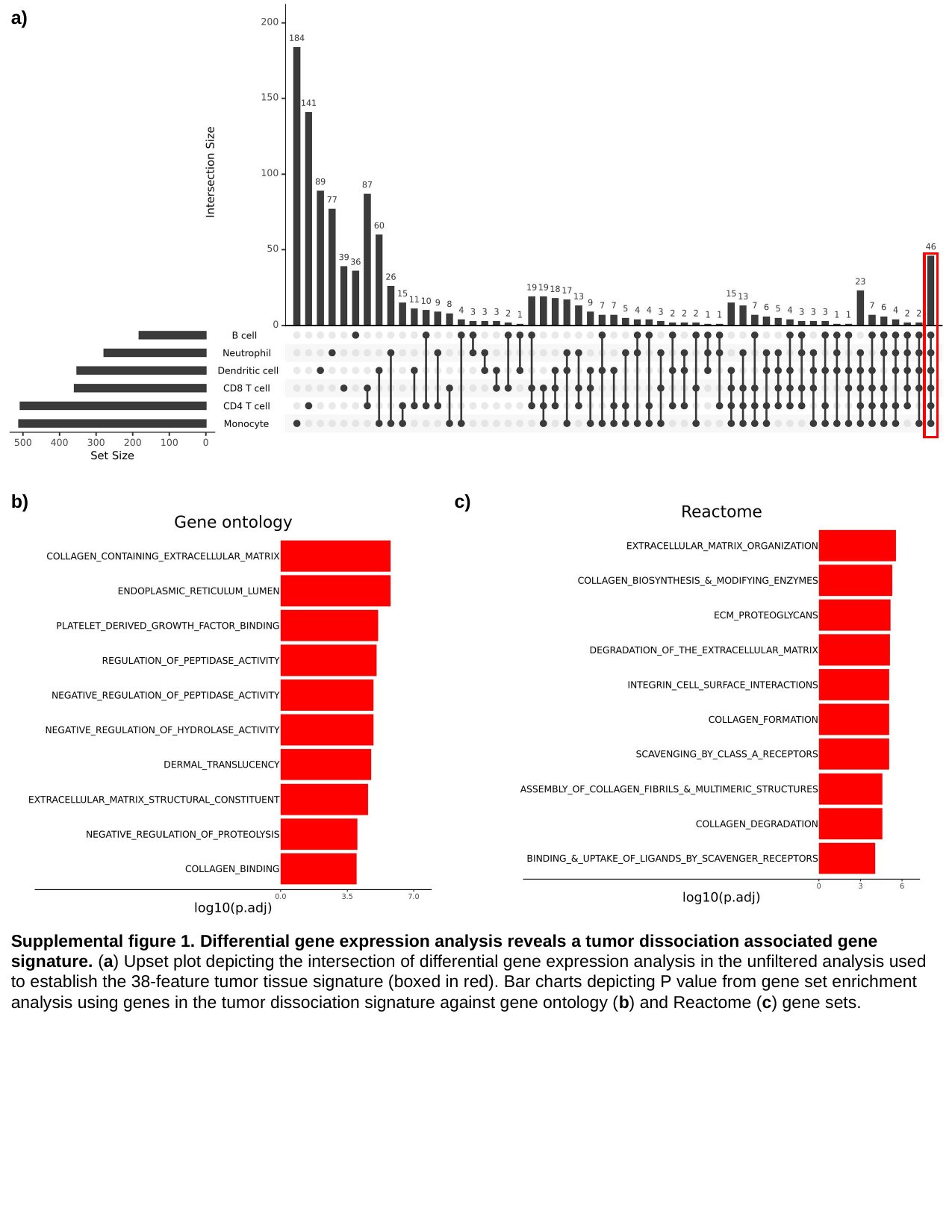

a)
b)
c)
Supplemental figure 1. Differential gene expression analysis reveals a tumor dissociation associated gene signature. (a) Upset plot depicting the intersection of differential gene expression analysis in the unfiltered analysis used to establish the 38-feature tumor tissue signature (boxed in red). Bar charts depicting P value from gene set enrichment analysis using genes in the tumor dissociation signature against gene ontology (b) and Reactome (c) gene sets.

## Slide 5
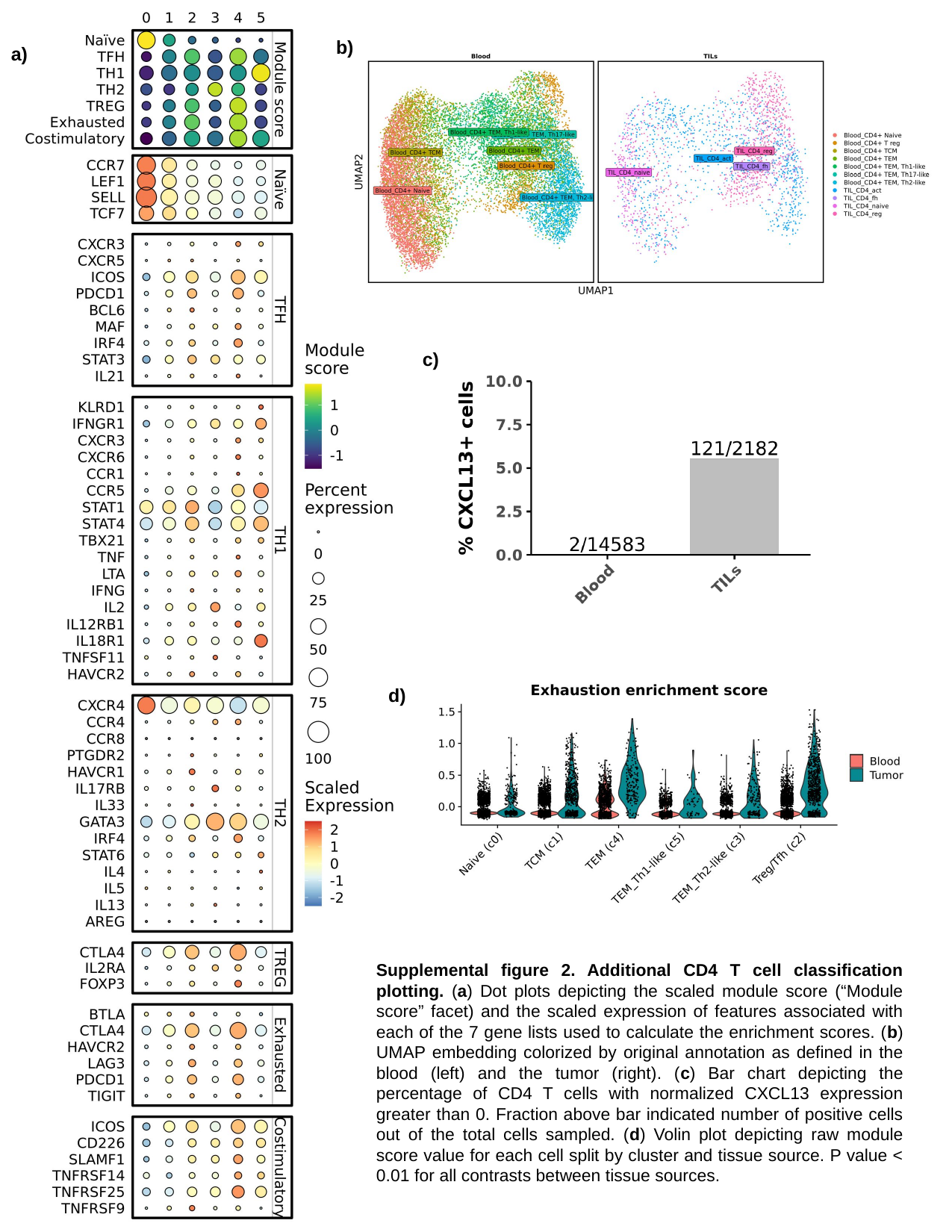

b)
a)
c)
d)
Supplemental figure 2. Additional CD4 T cell classification plotting. (a) Dot plots depicting the scaled module score (“Module score” facet) and the scaled expression of features associated with each of the 7 gene lists used to calculate the enrichment scores. (b) UMAP embedding colorized by original annotation as defined in the blood (left) and the tumor (right). (c) Bar chart depicting the percentage of CD4 T cells with normalized CXCL13 expression greater than 0. Fraction above bar indicated number of positive cells out of the total cells sampled. (d) Volin plot depicting raw module score value for each cell split by cluster and tissue source. P value < 0.01 for all contrasts between tissue sources.

## Slide 6
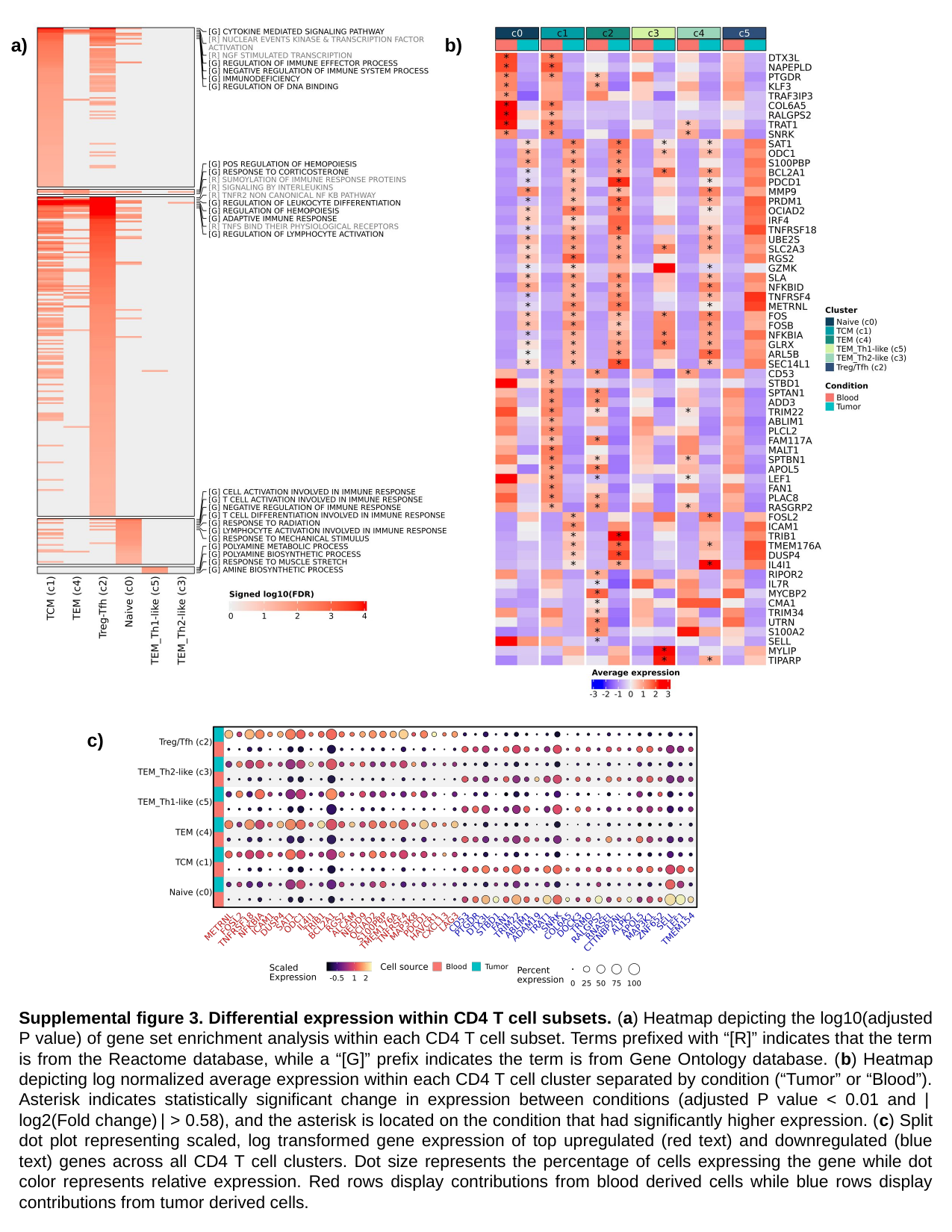

a)
b)
c)
Supplemental figure 3. Differential expression within CD4 T cell subsets. (a) Heatmap depicting the log10(adjusted P value) of gene set enrichment analysis within each CD4 T cell subset. Terms prefixed with “[R]” indicates that the term is from the Reactome database, while a “[G]” prefix indicates the term is from Gene Ontology database. (b) Heatmap depicting log normalized average expression within each CD4 T cell cluster separated by condition (“Tumor” or “Blood”). Asterisk indicates statistically significant change in expression between conditions (adjusted P value < 0.01 and | log2(Fold change) | > 0.58), and the asterisk is located on the condition that had significantly higher expression. (c) Split dot plot representing scaled, log transformed gene expression of top upregulated (red text) and downregulated (blue text) genes across all CD4 T cell clusters. Dot size represents the percentage of cells expressing the gene while dot color represents relative expression. Red rows display contributions from blood derived cells while blue rows display contributions from tumor derived cells.

## Slide 7
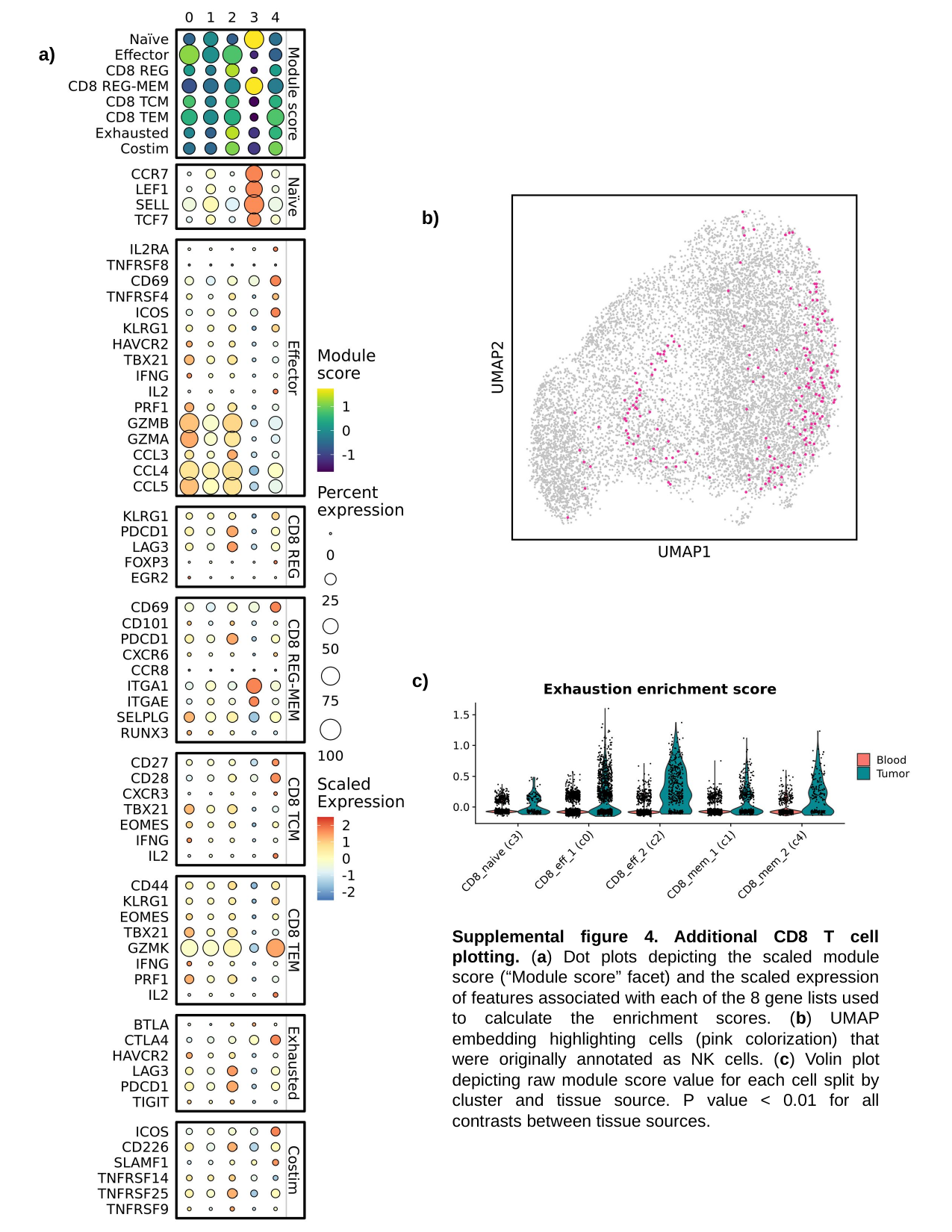

a)
b)
c)
Supplemental figure 4. Additional CD8 T cell plotting. (a) Dot plots depicting the scaled module score (“Module score” facet) and the scaled expression of features associated with each of the 8 gene lists used to calculate the enrichment scores. (b) UMAP embedding highlighting cells (pink colorization) that were originally annotated as NK cells. (c) Volin plot depicting raw module score value for each cell split by cluster and tissue source. P value < 0.01 for all contrasts between tissue sources.

## Slide 8
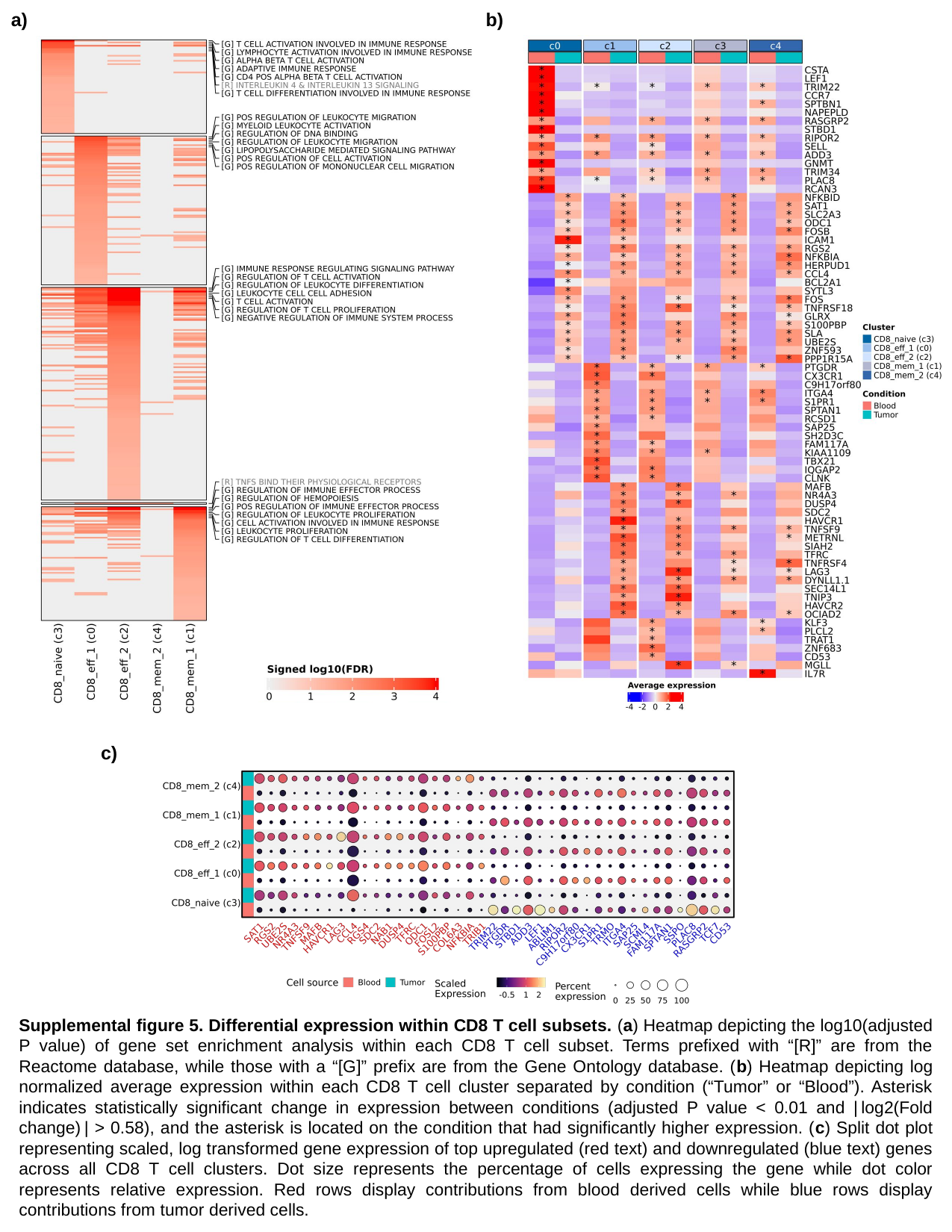

a)
b)
c)
Supplemental figure 5. Differential expression within CD8 T cell subsets. (a) Heatmap depicting the log10(adjusted P value) of gene set enrichment analysis within each CD8 T cell subset. Terms prefixed with “[R]” are from the Reactome database, while those with a “[G]” prefix are from the Gene Ontology database. (b) Heatmap depicting log normalized average expression within each CD8 T cell cluster separated by condition (“Tumor” or “Blood”). Asterisk indicates statistically significant change in expression between conditions (adjusted P value < 0.01 and | log2(Fold change) | > 0.58), and the asterisk is located on the condition that had significantly higher expression. (c) Split dot plot representing scaled, log transformed gene expression of top upregulated (red text) and downregulated (blue text) genes across all CD8 T cell clusters. Dot size represents the percentage of cells expressing the gene while dot color represents relative expression. Red rows display contributions from blood derived cells while blue rows display contributions from tumor derived cells.

## Slide 9
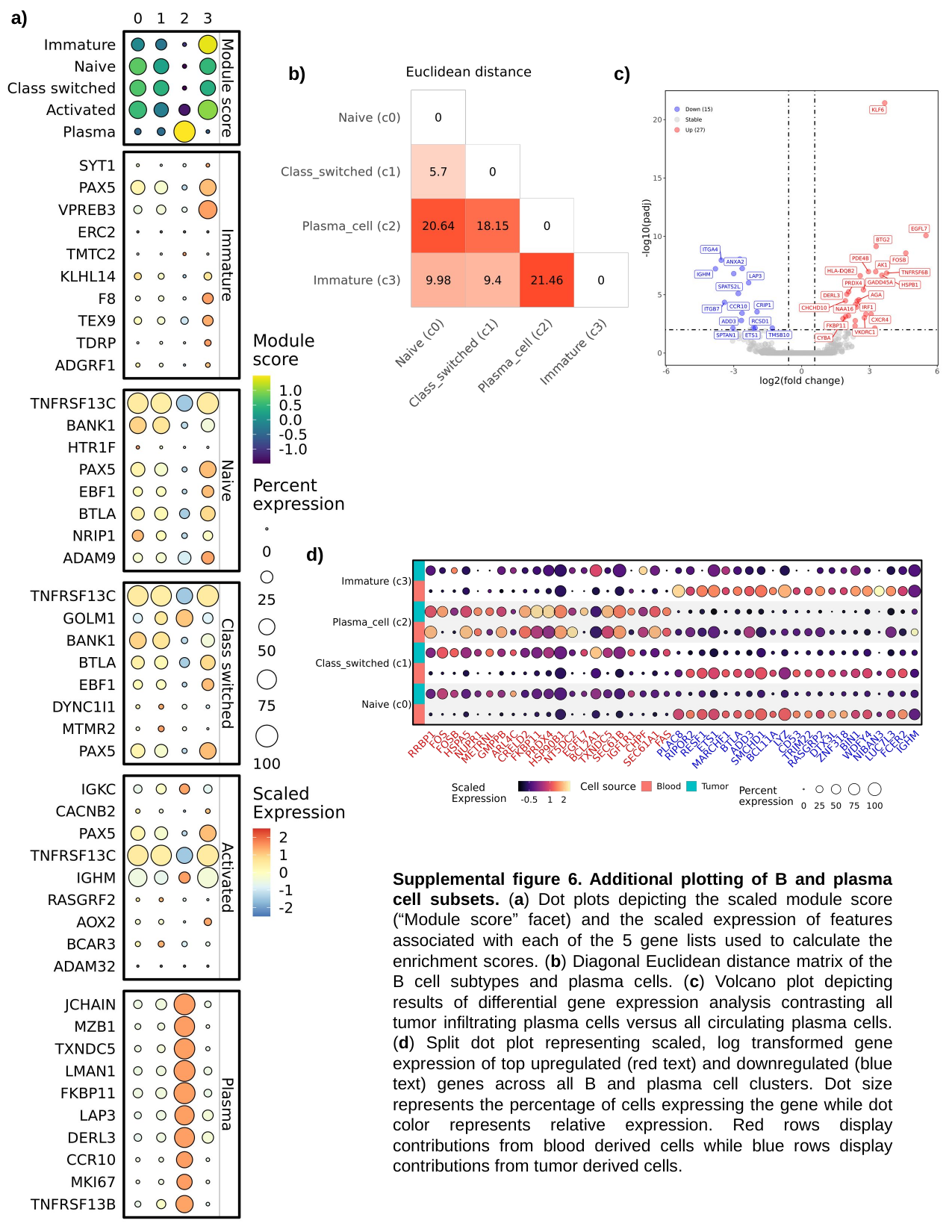

a)
b)
c)
d)
Supplemental figure 6. Additional plotting of B and plasma cell subsets. (a) Dot plots depicting the scaled module score (“Module score” facet) and the scaled expression of features associated with each of the 5 gene lists used to calculate the enrichment scores. (b) Diagonal Euclidean distance matrix of the B cell subtypes and plasma cells. (c) Volcano plot depicting results of differential gene expression analysis contrasting all tumor infiltrating plasma cells versus all circulating plasma cells. (d) Split dot plot representing scaled, log transformed gene expression of top upregulated (red text) and downregulated (blue text) genes across all B and plasma cell clusters. Dot size represents the percentage of cells expressing the gene while dot color represents relative expression. Red rows display contributions from blood derived cells while blue rows display contributions from tumor derived cells.

## Slide 10
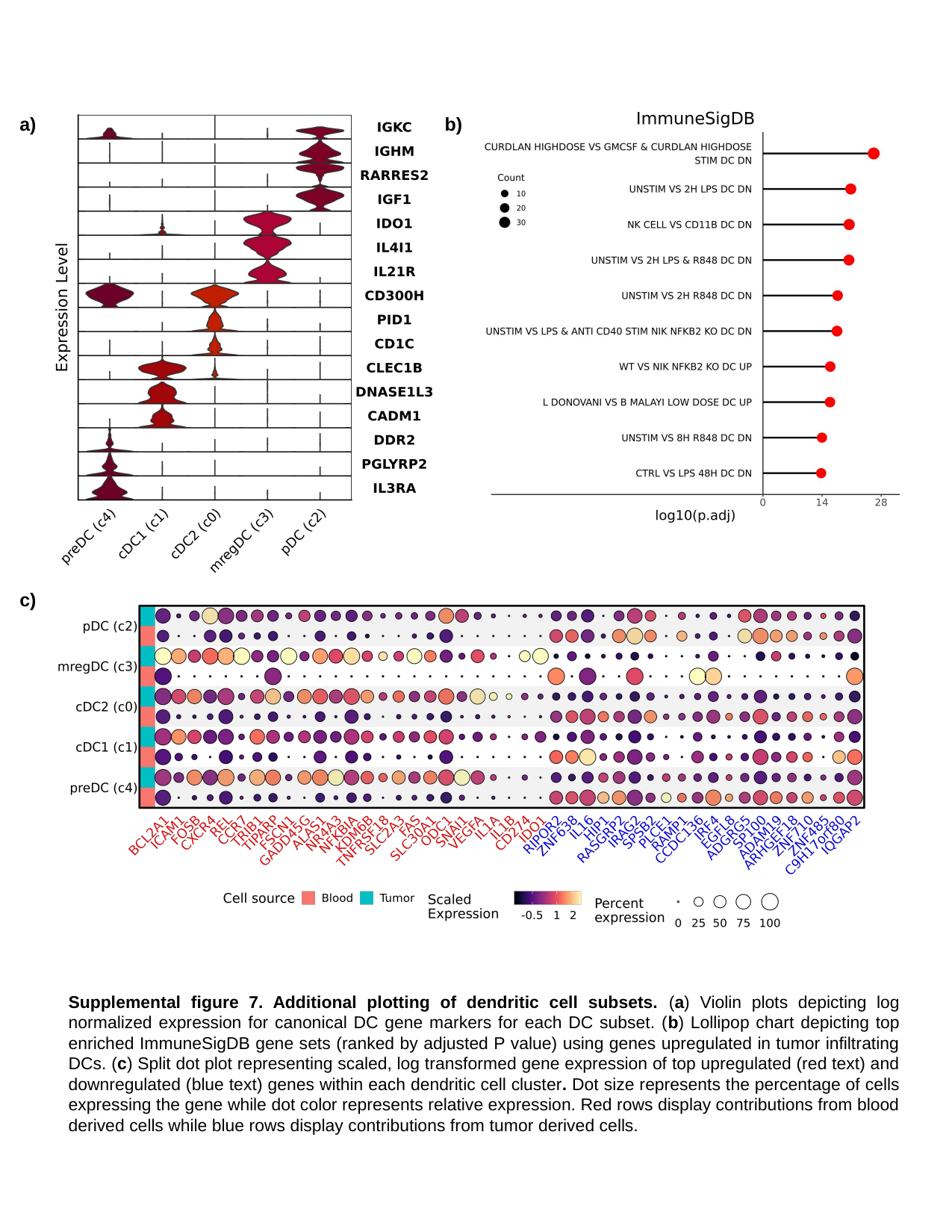

b)
a)
c)
Supplemental figure 7. Additional plotting of dendritic cell subsets. (a) Violin plots depicting log normalized expression for canonical DC gene markers for each DC subset. (b) Lollipop chart depicting top enriched ImmuneSigDB gene sets (ranked by adjusted P value) using genes upregulated in tumor infiltrating DCs. (c) Split dot plot representing scaled, log transformed gene expression of top upregulated (red text) and downregulated (blue text) genes within each dendritic cell cluster. Dot size represents the percentage of cells expressing the gene while dot color represents relative expression. Red rows display contributions from blood derived cells while blue rows display contributions from tumor derived cells.

## Slide 11
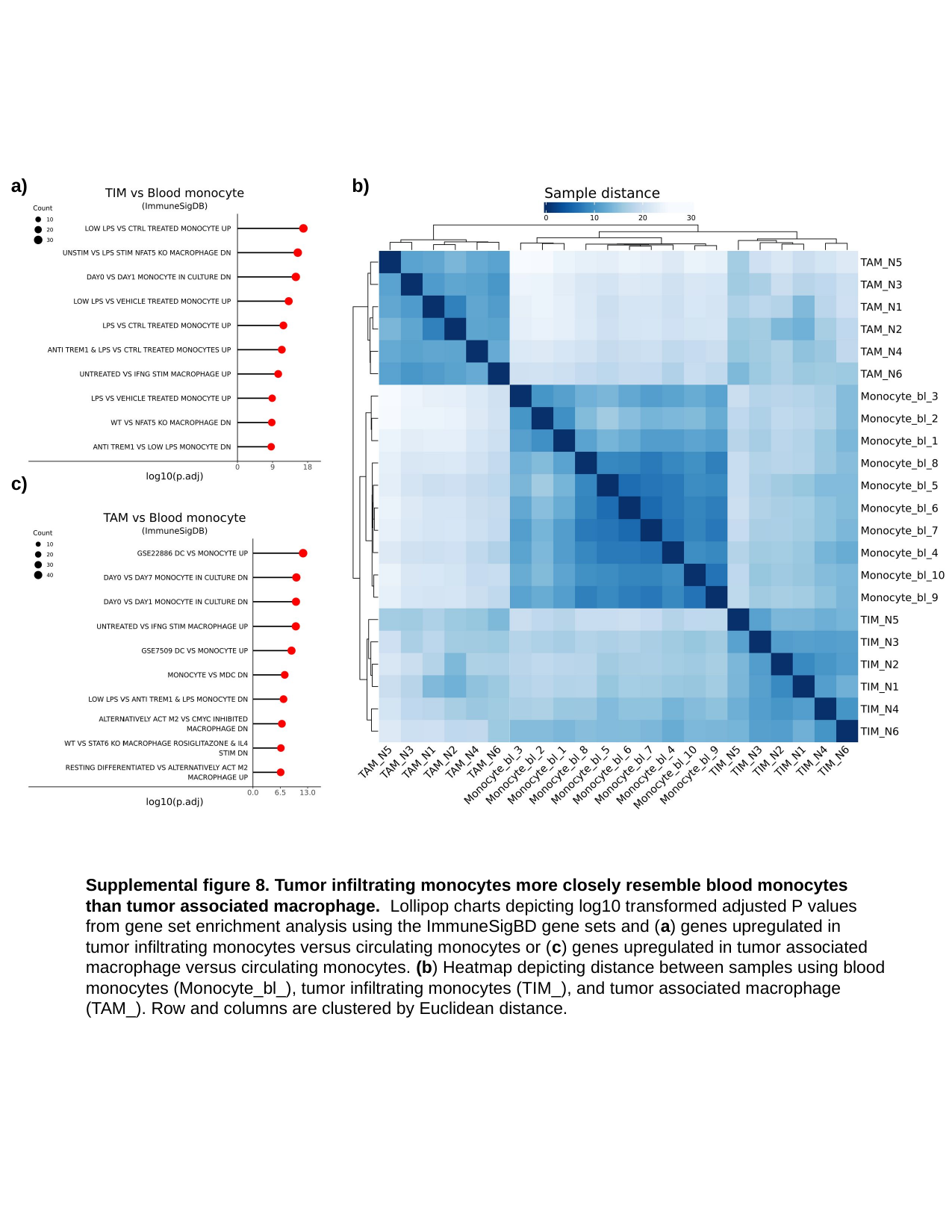

b)
a)
c)
Supplemental figure 8. Tumor infiltrating monocytes more closely resemble blood monocytes than tumor associated macrophage. Lollipop charts depicting log10 transformed adjusted P values from gene set enrichment analysis using the ImmuneSigBD gene sets and (a) genes upregulated in tumor infiltrating monocytes versus circulating monocytes or (c) genes upregulated in tumor associated macrophage versus circulating monocytes. (b) Heatmap depicting distance between samples using blood monocytes (Monocyte_bl_), tumor infiltrating monocytes (TIM_), and tumor associated macrophage (TAM_). Row and columns are clustered by Euclidean distance.

## Slide 12
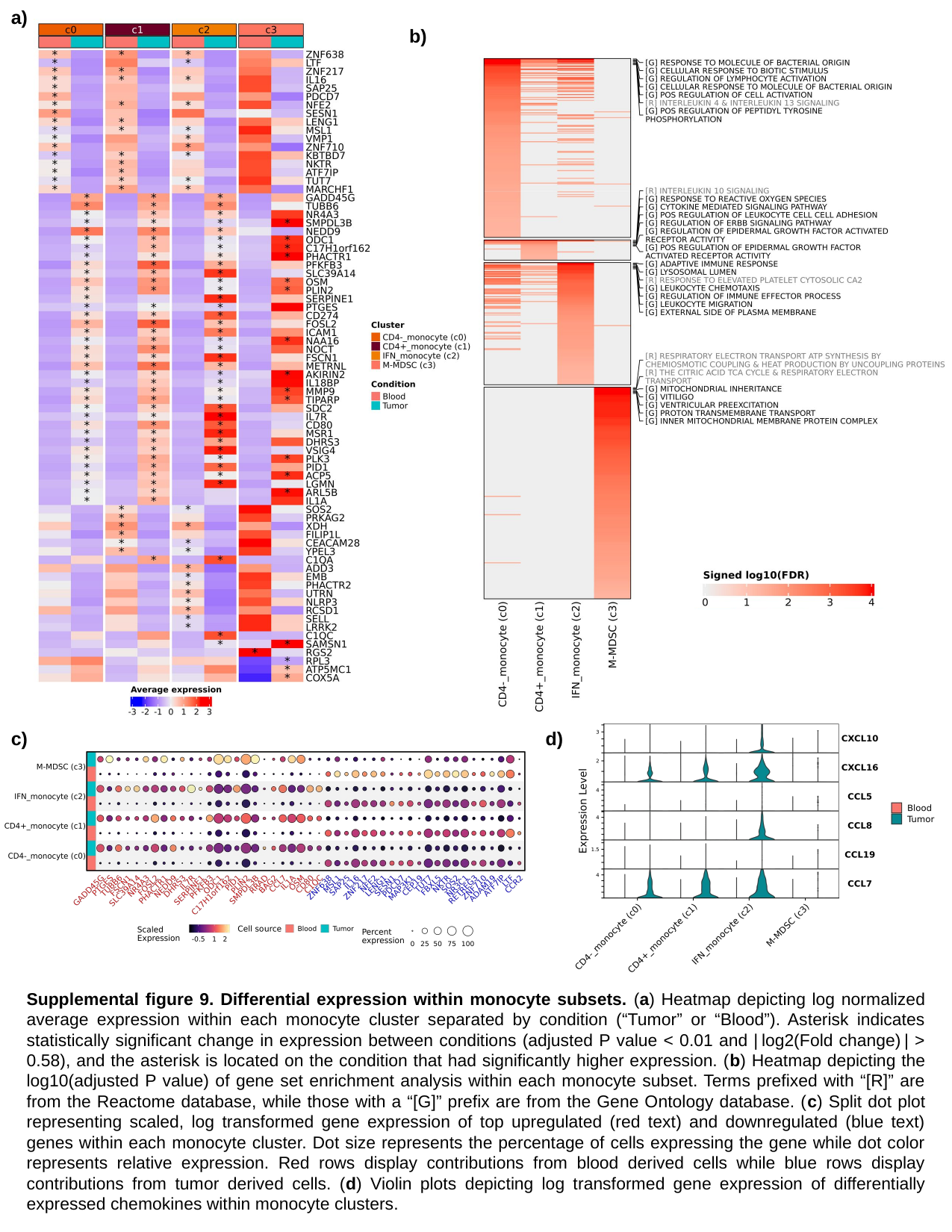

a)
b)
c)
d)
Supplemental figure 9. Differential expression within monocyte subsets. (a) Heatmap depicting log normalized average expression within each monocyte cluster separated by condition (“Tumor” or “Blood”). Asterisk indicates statistically significant change in expression between conditions (adjusted P value < 0.01 and | log2(Fold change) | > 0.58), and the asterisk is located on the condition that had significantly higher expression. (b) Heatmap depicting the log10(adjusted P value) of gene set enrichment analysis within each monocyte subset. Terms prefixed with “[R]” are from the Reactome database, while those with a “[G]” prefix are from the Gene Ontology database. (c) Split dot plot representing scaled, log transformed gene expression of top upregulated (red text) and downregulated (blue text) genes within each monocyte cluster. Dot size represents the percentage of cells expressing the gene while dot color represents relative expression. Red rows display contributions from blood derived cells while blue rows display contributions from tumor derived cells. (d) Violin plots depicting log transformed gene expression of differentially expressed chemokines within monocyte clusters.

## Slide 13
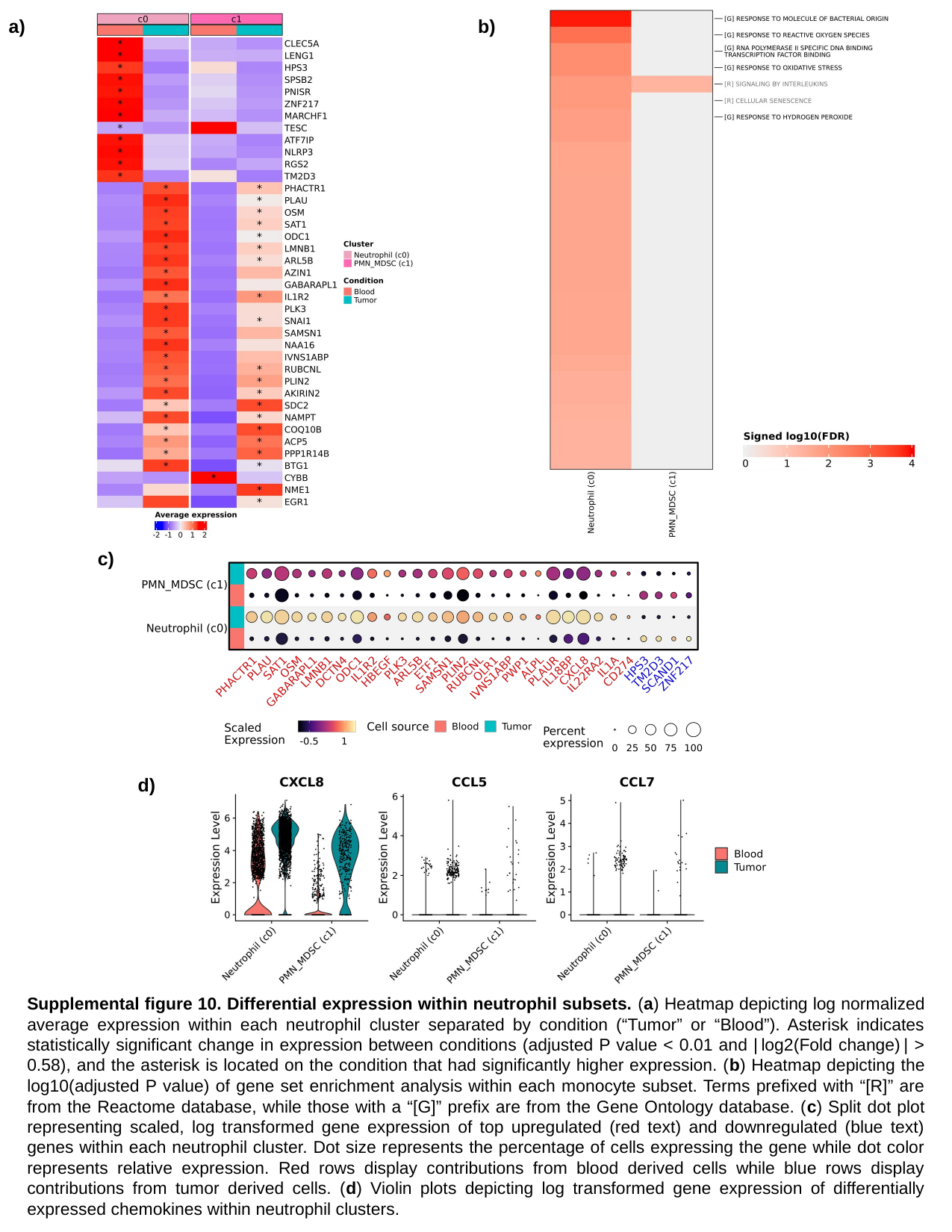

b)
a)
c)
d)
Supplemental figure 10. Differential expression within neutrophil subsets. (a) Heatmap depicting log normalized average expression within each neutrophil cluster separated by condition (“Tumor” or “Blood”). Asterisk indicates statistically significant change in expression between conditions (adjusted P value < 0.01 and | log2(Fold change) | > 0.58), and the asterisk is located on the condition that had significantly higher expression. (b) Heatmap depicting the log10(adjusted P value) of gene set enrichment analysis within each monocyte subset. Terms prefixed with “[R]” are from the Reactome database, while those with a “[G]” prefix are from the Gene Ontology database. (c) Split dot plot representing scaled, log transformed gene expression of top upregulated (red text) and downregulated (blue text) genes within each neutrophil cluster. Dot size represents the percentage of cells expressing the gene while dot color represents relative expression. Red rows display contributions from blood derived cells while blue rows display contributions from tumor derived cells. (d) Violin plots depicting log transformed gene expression of differentially expressed chemokines within neutrophil clusters.

## Slide 14
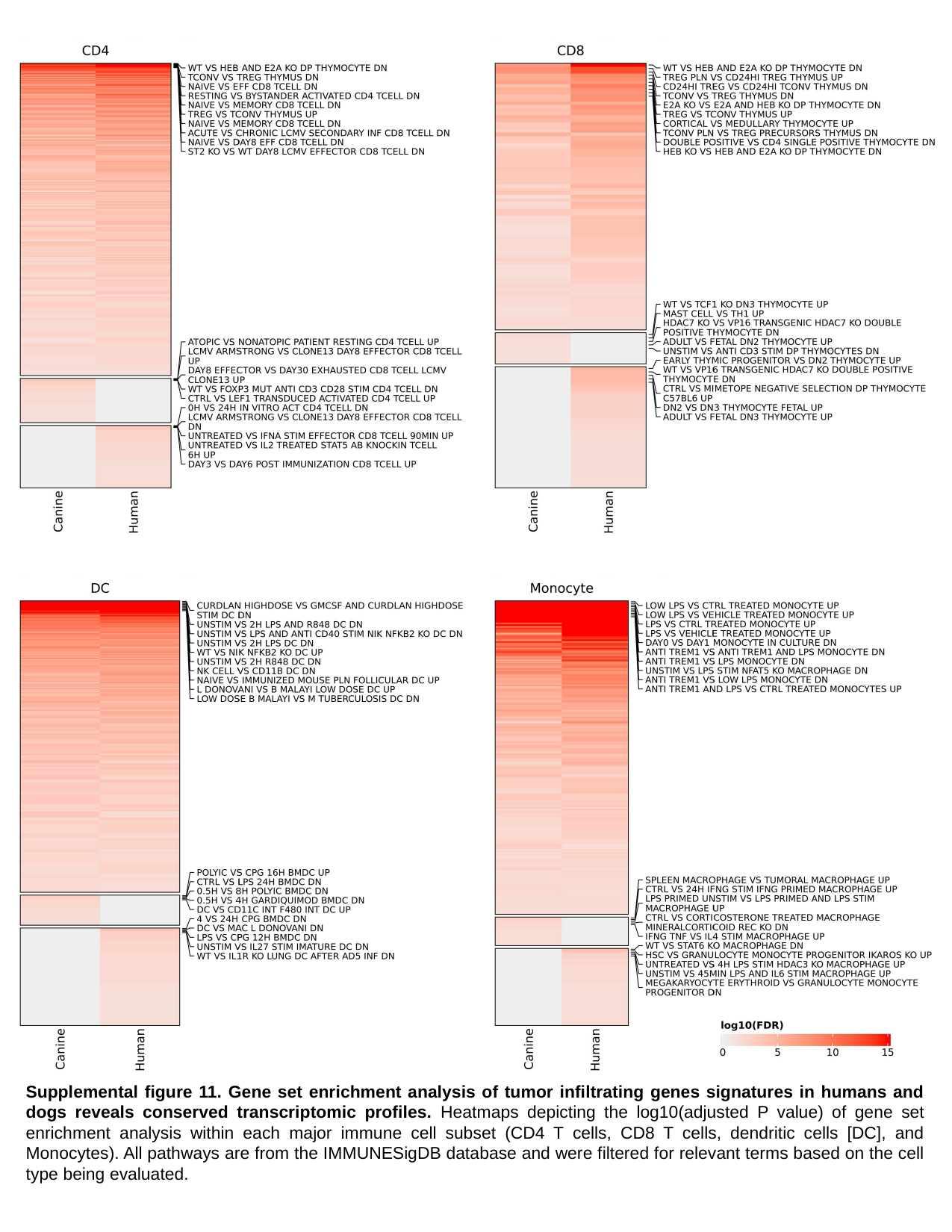

Supplemental figure 11. Gene set enrichment analysis of tumor infiltrating genes signatures in humans and dogs reveals conserved transcriptomic profiles. Heatmaps depicting the log10(adjusted P value) of gene set enrichment analysis within each major immune cell subset (CD4 T cells, CD8 T cells, dendritic cells [DC], and Monocytes). All pathways are from the IMMUNESigDB database and were filtered for relevant terms based on the cell type being evaluated.
